# Schizophrenia-like phenotypes in mice with NMDA receptor ablation in intralaminar thalamic nucleus cells and gene therapy-based reversal in adults

**DOI:** 10.1038/tp.2017.19

**Published:** 2017-02-28

**Authors:** K Yasuda, Y Hayashi, T Yoshida, M Kashiwagi, N Nakagawa, T Michikawa, M Tanaka, R Ando, A Huang, T Hosoya, T J McHugh, M Kuwahara, S Itohara

**Affiliations:** 1 Laboratory for Behavioral Genetics, RIKEN Brain Science Institute, Saitama, Japan; 2 Department of Veterinary Pathophysiology and Animal Health, Graduate School of Agricultural and Life Science, The University of Tokyo, Tokyo, Japan; 3 International Institute for Integrative Sleep Medicine (WPI-IIIS), University of Tsukuba, Ibaraki, Japan; 4 Laboratory for Local Neuronal Circuits, RIKEN Brain Science Institute, Saitama, Japan; 5 Biotechnological Optics Research Team, RIKEN Center for Advanced Photonics, Saitama, Japan; 6 Laboratory for Neuron-Glia Circuitry, RIKEN Brain Science Institute, Saitama, Japan; 7 Laboratory for Circuit and Behavioral Physiology, RIKEN Brain Science Institute, Saitama, Japan

## Abstract

In understanding the mechanism of schizophrenia pathogenesis, a significant finding is that drug abuse of phencyclidine or its analog ketamine causes symptoms similar to schizophrenia. Such drug effects are triggered even by administration at post-adolescent stages. Both drugs are *N*-methyl-d-aspartate receptor (NMDAR) antagonists, leading to a major hypothesis that glutamate hypofunction underlies schizophrenia pathogenesis. The precise region that depends on NMDAR function, however, is unclear. Here, we developed a mouse strain in which NMDARs in the intralaminar thalamic nuclei (ILN) were selectively disrupted. The mutant mice exhibited various schizophrenia-like phenotypes, including deficits in working memory, long-term spatial memory, and attention, as well as impulsivity, impaired prepulse inhibition, hyperlocomotion and hyperarousal. The electroencephalography analysis revealed that the mutant mice had a significantly reduced power in a wide range of frequencies including the alpha, beta and gamma bands, both during wake and rapid eye movement (REM) sleep, and a modest decrease of gamma power during non-REM sleep. Notably, restoring NMDARs in the adult ILN rescued some of the behavioral abnormalities. These findings suggest that NMDAR dysfunction in the ILN contributes to the pathophysiology of schizophrenia-related disorders. Furthermore, the reversal of inherent schizophrenia-like phenotypes in the adult mutant mice supports that ILN is a potential target site for a therapeutic strategy.

## Introduction

Schizophrenia is a disabling mental disorder, and core aspects are resistant to medications.^1^ Current treatments using dopamine D2 receptor blockers effectively treat psychotic symptoms such as hallucination and delusion, but have limited effect on negative symptoms^2^ and cognitive impairments (working memory, long-term memory, attention, impulsivity and perception deficits).^3, 4^ Genetic and environmental susceptibility factors linked to the disorder could influence postnatal brain maturation.^5, 6^ Neurodevelopmental models of schizophrenia suggest that schizophrenia is mainly a progressive and irreversible disease.^7^ Thus, there is an urgent need to develop animal models based on new insights gained from human studies to search for alternative therapeutic strategies.

Administration of *N*-methyl-d-aspartate receptor (NMDAR) antagonists such as phencyclidine or ketamine at post-adolescent stages in humans or rodents,^8, 9, 10, 11^ induces the full range of psychotic, negative and cognitive symptoms, suggesting that NMDAR dysfunction in the mature brain contributes to the pathology of schizophrenia.^12^ In further support of the NMDAR theory, a number of genes involved in glutamatergic signaling and synaptic plasticity were tagged by a large-scale schizophrenia genome-wide association study.^13^ Further, postmortem neurochemical studies of schizophrenia patients reveal reduced NMDAR expression in the thalamus,^14^ prefrontal cortex^15^ and hippocampus.^16^ Genetic manipulations that alter NMDAR subunit proteins also cause schizophrenia-like symptoms in mice.^17, 18, 19, 20^ Why disrupting NMDAR function leads to such symptoms and whether such effects can be explained by malfunction in a specific brain area, however, has remained unclear. Deletion of NMDARs in mature cortical excitatory neurons has little effect on cognitive symptoms.^18^ Thus, post-adolescent NMDAR hypofunction in the cortex does not fully account for all of the cognitive deficits in schizophrenia or the acute effects of NMDAR antagonists. NMDAR hypofunction in cortical GABAergic interneurons during early development, however, causes schizophrenia-like phenotypes,^19, 20^ supporting the developmental theory of schizophrenia.

Dysfunction of the thalamocortical networks may partially underlie the pathology in schizophrenia.^21, 22^ Neuroimaging studies of patients with schizophrenia suggest differences in the morphology and metabolism of the thalamic subnuclei, including the intralaminar thalamic nuclei (ILN).^23, 24^ Although the precise function of these nuclei in cognitive function is poorly understood,^25, 26^ some ILN neurons have rich reciprocal connections with the prefrontal cortex and striatum^27^—key structures involved in the control of cognitive function.^28, 29^ Such thalamocortical connectivity from the ILN to the prefrontal cortex is altered in patients with schizophrenia.^30, 31^ In addition, NMDAR expression in the ILN is reduced in patients with schizophrenia.^32^ These findings led us to hypothesize that NMDAR hypofunction in the ILN is causally related to the cognitive impairments observed in schizophrenia.

To test this hypothesis, we generated ILN-selective conditional knockout (cKO) mice for NR1, which encodes an essential NMDAR subunit. Comprehensive behavioral examination of the cKO mice revealed abnormalities resembling the symptoms of schizophrenia. We further tested whether the abnormalities could be rescued by viral vector-mediated restoration of NR1 in the adult ILN. Our findings support the notion that NMDAR hypofunction in the post-adolescent ILN has a crucial role in the pathophysiology of schizophrenia.

## Materials and methods

All the experiments were carried out in accordance with the Guide for the Care and Use of Laboratory Animals of the National Institute of Health. The experimental protocol was approved by the RIKEN institutional animal use committee.

### Animal

The mice were housed in individually ventilated cages in groups of two to five animals. The light cycle was 0800 h ON and 2000 h OFF. Water and food were provided *ad libitum*, unless otherwise stated. A total of 127 male and 60 female mice were used for histological, electrophysiological and behavior experiments. For Y-maze and Morris water maze tests, and EEG (electroencephalography) recording, the male mice were tested as described in each figure legend.

### Generation of ILN-specific NMDAR-deficient mice

A bacterial artificial chromosome clone (RP23-116A1) containing the *Lypd6b* (*LY6/PLAUR domain containing 6B*) gene was used to generate an ILN-specific Cre transgenic mouse line. The ILN-Cre mice were crossed with mice carrying the loxP-flanked *Grin1* allele^33^ to obtain ILN-specific NMDAR-deficient mice. All these mice had been maintained before use in C57BL/6J isogenic or congenic backgrounds.

### Viral injection for NMDAR rescue

For virus-mediated rescue of NR1 in the ILN of cKO mice, we used the AAV-fsNR1 virus,^34^ which expresses *Grin1* after Cre-mediated recombination (pAAV-fsNR1 provided by Dr Richard D Palmiter at the University of Washington).

### Behavioral analysis

All behavioral tasks were performed, as previously described,^19, 35^ during the light phase, between ZT2 and ZT12.

### Statistical analysis

Data were analyzed with Excel Statistics (Excel Toukei 2012, Social Survey Research Information), SPSS (SPSS Japan, Tokyo, Japan), MATLAB (Mathworks, Natick, MA, USA) and R (version 3.2.3). Mean differences between groups were analyzed using an unpaired two-sided *t*-test; one-way, two-way, three-way or mixed between–within-subjects analysis of variance, followed by Tukey’s *post hoc* tests. Normality was tested using the Kolmogorov–Smirnov test, and equality of variances was tested using Levene’s test. For nonparametric statistics, the Wilcoxon rank-sum test or Kruskal–Wallis test and Steel–Dwass multiple comparison tests were used. A *P*-value <0.05 was considered statistically significant.

The experimental details are provided in the Supplementary Information.

## Results

### Generation of the ILN-Cre transgenic mouse

**Figure 1:**
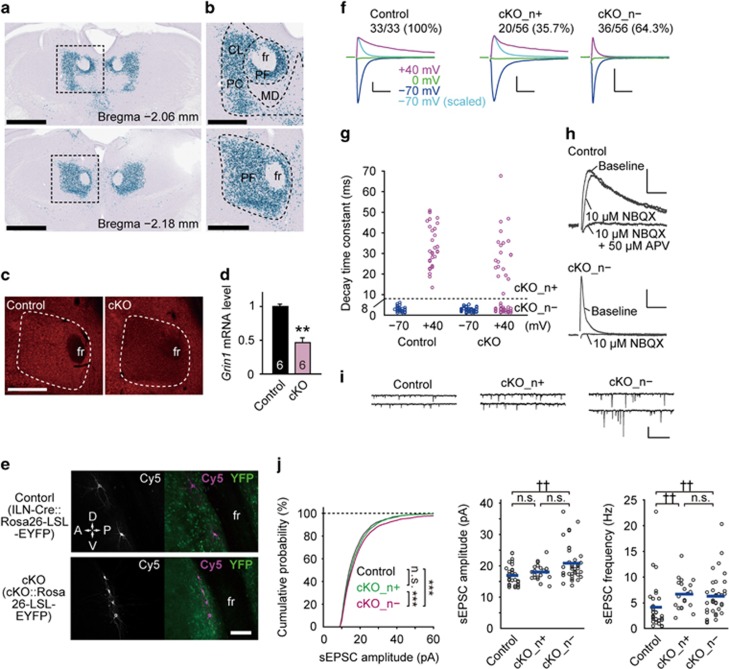
Generation of ILN neuron-selective NMDAR cKO mice. (**a** and **b**) Representative images of the spatial distribution of Cre recombinase activity in coronal sections from an ILN-Cre::Rosa-NLSLacZ (*Lypd6b-Cre*::*Gt(ROSA)26Sor*^*tm1Ito*^) double-transgenic mouse stained with X-gal (blue) and hematoxylin (purple). (**c**) Representative immunohistochemistry images for NR1 in 6-month-old control NR1-flox (*Grin1*^*flox/flox*^) and cKO (ILN-NR1-cKO) mice. (**d**) Quantitative RT-PCR for *Grin1* mRNA in the ILN of control and cKO mice (six samples (three females) for each group, 2 months old). (**e**) Representative confocal images of recorded cells (Cy5 labeled) after whole-cell patch-clamp recordings. (**f**) EPSCs recorded at the holding potential of −70 mV (blue), +40 mV (magenta) and 0 mV (green). EPSCs recorded at −70 mV that were scaled to the peak of EPSCs recorded at +40 mV are also shown for comparison of the EPSC time course (light blue). cKO_n+ and cKO_n−, cKO neuron with and without NMDA current, respectively. Scale bars, 10 ms and 100 pA. (**g**) Decay time constant of EPSCs recorded at −70 mV and +40 mV in individual cells. Control, *n*=33; cKO, *n*=56. Existence of NMDA currents was judged at the time constant of 8 ms (gray broken line). (**h**) Blockade of EPSCs recorded at +40 mV by AMPA receptor blocker NBQX and NMDA receptor blocker APV. Scale bars, 10 ms, 20 pA (Control) and 50 pA (cKO_n−). (**i**) Left, sEPSCs recorded at −70 mV. Scale bars, 0.5 s and 40 pA. (**j**) Left: cumulative probability of amplitude of sEPSC. Seventy-five random events were selected from individual cells and events from control, cKO_n+ and cKO_n− cells were respectively pooled. Center and right: amplitude and frequency of sEPSCs in individual cells (circle) and mean values (horizontal bar, Control, *n*=33; cKO_n+, *n*=20; cKO_n−, *n*=36). **P<0.01 (Wilcoxon rank-sum test). ***P<0.001; NS, not significant (Kolmogorov–Smirnov test). ^††^*P*<0.01 (Kruskal–Wallis test, *post hoc* Steel–Dwass multiple comparison test). Scale bars, 1 mm (**a**), 500 μm (**b** and **c**), 100 μm (**e**). All error bars represent s.e.m. cKO, conditional knockout; CL, centrolateral thalamic nucleus; EPSC, excitatory postsynaptic current; fr, fasciculus retroflexus; ILN, intralaminar thalamic nuclei; MD, mediodorsal thalamic nucleus; NMDAR, *N*-methyl-d-aspartate receptor; PC, paracentral thalamic nucleus; PF, parafascicular thalamic nucleus.

To achieve genetic manipulation specifically in the ILN, we generated transgenic mouse lines *Lypd6b-Cre* in which the Cre recombinase expression was selectively induced in the ILN, including the parafascicular, centrolateral and paracentral subnuclei (Figures 1a and b). The LacZ-positive cells representing Cre-mediated recombination comprised 87.3±2.2% of the NeuN-positive cells in the ILN (Supplementary Figure 1). Smaller numbers of LacZ-expressing neurons were also detected in the mediodorsal, central medial and reuniens nuclei of the thalamus, cortex, hippocampus, superior colliculus and medulla (Supplementary Figure 2). Cre expression began at embryonic day 18, and reached the adult level by postnatal day 21.

### Ablation of NMDARs in ILN cells

*Lypd6b-Cre* (ILN-Cre) mice were crossed with *Grin1*^*flox/flox*^ mice,^33^ which encode NR1, to generate ILN-NR1-cKO mice. Immunohistochemistry for NR1, an essential subunit of the NMDA receptor, revealed a marked selective decrease in the ILN (Figure 1c). *Grin1* mRNA levels determined by quantitative reverse transcription-polymerase chain reaction (RT-PCR) in tissue samples containing the ILN were reduced by 54% in cKO mice compared with control samples (Figure 1d).

We confirmed the functional loss of NMDARs by whole-cell patch-clamp recording. Cre-positive cells were visualized by crossing ILN-NR1-cKO or control ILN-Cre mice with a loxP-flanked enhanced yellow fluorescent protein line. Recordings were performed on enhanced yellow fluorescent protein(+) cells at around postnatal 4 weeks (Figure 1e). In all 33 cells tested from five control animals, electrical stimulation-induced excitatory postsynaptic currents (EPSCs) recorded at a holding potential of +40 mV had a longer decay time constant than those recorded at −70 mV (Figures 1f and g). EPSCs recorded at +40 mV were partially blocked by the α-amino-3-hydroxy-5-methyl-4-isoxazolepropionic acid (AMPA) receptor blocker NBQX, and completely blocked by additional application of the NMDA receptor blocker APV (Figure 1h, top). In contrast, for the cKO mice, in 36 of 56 cells (64.3%) tested from seven animals, the decay time constants of EPSCs recorded at +40 and −70 mV were almost identical, both less than 8 ms (Figures 1f and g; cKO_n− cells). In these cells, application of NBQX alone completely blocked EPSCs recorded at +40 mV (Figure 1h, bottom). These findings confirmed that NMDARs were functionally eliminated from the majority of Cre-targeted ILN cells. We also analyzed spontaneous EPSCs (sEPSCs) recorded from ILN neurons that showed or did not show NMDA currents by electrical stimulation in cKO mice (cKO_n+ or cKO_n−, respectively) and sEPSCs recorded from ILN neurons in control mice (Figure 1i). For the amplitudes of sEPSCs, ILN neurons of cKO_n− had larger amplitudes compared with other groups (Figure 1j, left and middle). On the other hand, sEPSC frequencies were larger in both ILN neurons of cKO_n+ and cKO_n− compared with those in control mice (Figure 1j, right). These results suggest that NMDAR signaling dysfunction leads to hyperactivity in the ILN circuitry by intrinsic and extrinsic mechanisms.

### Cognitive impairments in ILN-NR1-cKO mice

**Figure 2:**
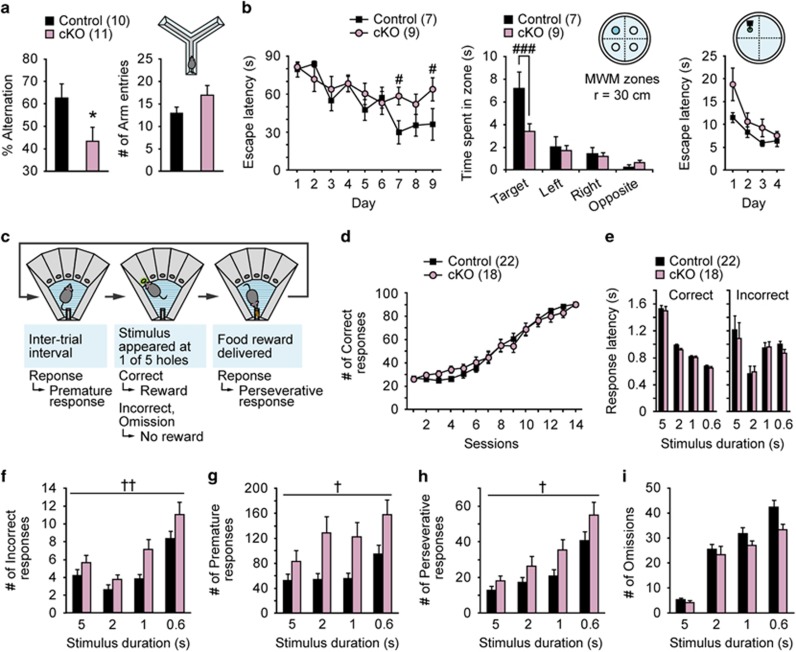
Schizophrenia-like cognitive impairments induced by ILN NMDAR deletion. (**a**) In the Y-maze spontaneous alternation task, cKO mice exhibited reduced alternation behavior. Number of arm entries did not significantly differ between genotypes (10 and 11 males, 2.5 months old). (**b**) Left: in the Morris water maze test, cKO mice required more time to locate the hidden platform in the acquisition phase of the water maze than control mice (seven and nine males, 6.5 months old). Center: during the probe test, cKO mice spent less time in the trained zones with a radius of 30 cm. Right: in the visible platform test, latency to escape did not differ significantly between the and cKO mice. (**c**) Schematic representation of the 5-CSRTT for attention and impulsivity. The operant chamber of the 5-CSRTT is equipped with five apertures that can be illuminated, and a tray to deliver a food reward. (**d**) The acquisition rate in the initial stage of the 5-CSRTT did not differ significantly between the mutant and control mice (22 controls (13 females and 9 males) and 18 mutants (9 females and 9 males), 8 months old). (**e**) No significant difference in the response speed of correct and incorrect responses was observed between genotypes. (**f**) Number of errors was increased in cKO mice. (**g**) Number of premature responses was increased in cKO mice. (**h**) Number of perseverative responses was increased in cKO mice. (**i**) No significant difference in the number of omission errors was detected between genotypes. *P<0.05 (unpaired *t*-test). ^#^*P*<0.05, ^###^*P*<0.001 (one-way analysis of variance (ANOVA)). ^†^*P*<0.05, ^††^*P*<0.01 (Mixed between–within-subjects ANOVA). All error bars represent s.e.m. cKO, conditional knockout; CSRTT, choice serial reaction time task; ILN, intralaminar thalamic nuclei; NMDAR, *N*-methyl-d-aspartate receptor.

Cognitive impairment is a core symptom of schizophrenia among psychiatric disorders.^3^ We assessed working memory in ILN-NR1-cKO mice using the Y-maze spontaneous alternation task. Control mice exhibited reliable alternation, whereas the cKO mice displayed reduced alternations (Figure 2a). The cKO mice and control mice had similar numbers of arm entries, suggesting that general activity levels were not altered (Figure 2a). These findings suggest that spatial working memory is impaired in cKO mice.

To assess long-term spatial reference memory, we tested the cKO mice in the Morris water maze. The cKO mice had longer latencies to find the hidden platform (Figure 2b). In the probe test, cKO mice spent less time swimming near the previous platform location compared with control mice, suggesting impaired memory (Figure 2b). The cKO mice had normal escape latencies when tested with a visible platform, indicating intact visual ability and motivation to escape (Figure 2b). These findings indicate that the cKO mice have deficits in spatial learning and memory.

To explore attention deficits in the cKO mice, we used the five-choice serial reaction time task.^35^ In this task, the animals are required to visually monitor five apertures and to identify, by a nose poke, which one is illuminated to obtain a reward (Figure 2c). No difference was detected between the control and cKO mice in learning speed (Figure 2d) or in the latency to make either a correct response or an incorrect response (Figure 2e). When the visual stimulus duration was shortened, however, the number of incorrect responses increased in cKO mice compared with control siblings (Figure 2f), suggesting that cKO mice had attention deficits. In addition, cKO mice had more premature responses (nose pokes made before the presentation of a target stimulus) and perseverative responses (continued nose pokes after a correct response and before the collection of the reward), which are measures of impulsiveness and compulsiveness,^35^ respectively (Figures 2g and h). No difference in the number of omissions (failure to make a response) was detected between the groups (Figure 2i). These findings suggest that NMDAR deletion in the ILN impairs attention and inhibitory control.

### Positive symptom-like behaviors in ILN-NR1-cKO mice

**Figure 3:**
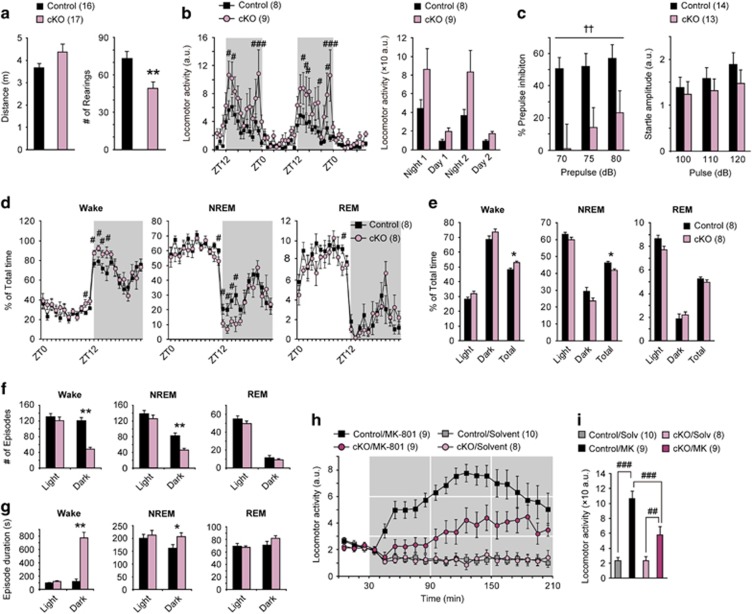
ILN NMDAR deletion leads to various other schizophrenia-like phenotypes. (**a**) Distance traveled following exposure to the novel open field did not differ significantly between genotypes. The number of rearings was decreased in cKO mice versus controls (16 controls (8 females and 8 males) and 17 cKO mice (8 females and 9 males), 3 months old). (**b**) Left: animals were placed in a home cage and locomotor activity was compared between control and cKO mice. Right: cumulative distance traveled during the night and day (eight and nine females, 3.5 months old). (**c**) Left: the percentage prepulse inhibition of cKO mice was significantly smaller than that of controls. Right: the startle amplitude did not differ significantly between genotypes. Data from females and males were pooled, as neither main effects of sex nor interaction effects between sex and genotype were detected (14 controls (6 females and 8 males) and 13 cKO mice (6 females and 7 males), 12 months old). (**d**) cKO mice exhibited increased wakefulness and reduced NREM sleep (eight control and eight mutant males, 4–6 months old). (**e**) Mean amount of sleep and wakefulness during the light or dark period and the whole day. (**f** and **g**) The number (**f**) and duration (**g**) of episodes of each sleep/wake stage in the control and cKO mice. During the dark phase, the duration of wake episodes was largely increased in cKO mice. (**h**) Time course of MK-801 induced hyperlocomotion in control and cKO mice. (**i**) Cumulative distance traveled after MK-801 treatment (30–210 min) was altered in cKO mice. The values are means±s.e.m. for 10 controls treated with saline (6 females and 4 males), nine controls treated with MK-801 (five females and four males), eight cKO mice treated with saline (five females and three males) and nine cKO treated with saline (five females and four males) at the age of 12 months. *P<0.05, **P<0.01 (unpaired *t*-test). ^#^*P*<0.05, ^##^*P*<0.01, ^###^*P*<0.001 (one-way analysis of variance (ANOVA)). ^††^*P*<0.01 (Mixed between–within-subjects ANOVA). All error bars represent s.e.m. cKO, conditional knockout; ILN, intralaminar thalamic nuclei; NMDAR, *N*-methyl-d-aspartate receptor; NREM, non-rapid eye movement.

Patients with schizophrenia exhibit positive and negative symptoms.^3, 4^ In rodent models, locomotor activity is widely used to assess positive symptom-like behaviors.^10^ Horizontal locomotor activity in cKO mice exposed to a novel open field did not differ significantly from that in control mice, whereas cKO mice exhibited less frequent rearing (vertical activity), which may reflect a reduction in general attention (Figure 3a).^36^ Home cage activity monitoring of the cKO mice for 2 days, however, revealed increased mean locomotor activity during the dark phase (Figure 3b). Prepulse inhibition is a measure of sensory filtering and is reduced in both patients with schizophrenia^4^ and rodent models of schizophrenia.^4, 10^ The cKO mice displayed impaired prepulse inhibition, whereas the auditory response itself was intact (Figure 3c). These findings, together with home cage hyperactivity, indicate that the cKO mice exhibit positive symptom-like behaviors. Moreover, as described above, cKO mice did not differ from control mice in exhibiting motivation to acquire a reward in the five-choice serial reaction time task.

### Increased arousal and abnormal sleep architecture in ILN-NR1-cKO mice

Patients with schizophrenia often experience sleep disruption due to reduced non-rapid eye movement (NREM) sleep, although the amount of REM sleep tends to be normal.^37^ The cKO mice exhibited decreased NREM sleep, which was replaced by increased wakefulness, whereas the amount of REM sleep was mostly unaffected (Figures 3d and e). This trend was most obvious at the beginning of the dark period, and no obvious sleep rebound was observed (Figure 3d). The episode duration of wake in the dark phase was dramatically increased in the cKO mice, suggesting a hyperarousal state (Figures 3f and g). To further examine the possibility that cKO mice had abnormally high arousal at the beginning of the dark period, the sleep/wake patterns were compared following a cage change, which is a well-established method for short-term sleep deprivation.^38^ The cKO mice exhibited increased wakefulness following a cage change during the dark period but not during the light period (Supplementary Figures 3a and b). Furthermore, although the amount of each sleep/wake state was indistinguishable between the control mice and cKO mice during the light period, the cKO mice displayed a shortened REM sleep latency (Supplementary Figure 3c), which is well recognized in schizophrenic patients.^37^ These results indicate that cKO mice exhibited enhanced arousal at the expense of NREM sleep in the beginning of dark phase, mimicking the hyperarousal state of schizophrenia, and an altered sleep architecture during the light phase also mimicking that of schizophrenia.^39^

### Altered sensitivity to the psychostimulant effects of MK-801 in ILN-NR1-cKO mice

Administration of an NMDA antagonist acutely and temporarily induces psychosis-like symptoms and hyperactivity in normal rodents.^10^ To examine whether the ILN is the primary site of action of the NMDA antagonists, we investigated the effects of the NMDAR antagonist MK-801 in the cKO mice. Subcutaneous administration of MK-801 induced locomotor hyperactivity that was sustained for over 3 h after injection in control animals. The MK-801-induced hyperactivity, however, was largely diminished in cKO mice (Figures 3h and i), suggesting that the ILN is a site of action of MK-801.

### Altered cortical oscillations in ILN-NR1-cKO mice

**Figure 4:**
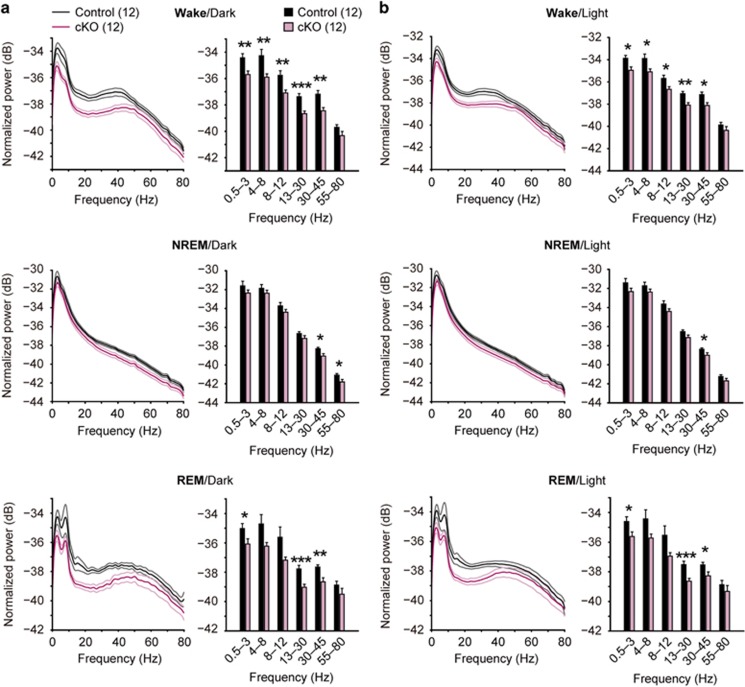
Altered neural oscillations in ILN-NR1-cKO mice. (**a**) Normalized power spectrum of cortical EEG recordings from control and cKO mice during the dark period ((six control and six mutant males) × 2 days, 4–6 months old). Top: decreased power in cKO mice of the 0.5–3 Hz delta, 4–8 Hz theta, 8–12 Hz alpha, 13–30 Hz beta and 30–45 Hz gamma frequency during wakefulness under the dark period. Middle: decreased power in cKO mice of the 30–45 Hz and 55–80 Hz gamma frequency during NREM sleep under the dark period. Bottom: decreased power in cKO mice of the 0.5–3 Hz delta, 13–30 Hz beta and 30–45 Hz gamma frequency during REM sleep under the dark period. (**b**) Normalized power spectrum for EEG recordings from control and cKO mice during the light period ((six control and six mutant males) × 2 days, 4–6 months old). Top: decreased power in cKO mice of the 0.5–3 Hz delta, 4–8 Hz theta, 8–12 Hz alpha, 13–30 Hz beta and 30–45 Hz gamma frequency during wakefulness under the light period. Middle: decreased power in cKO mice of the 30–45 Hz gamma frequency during NREM sleep under the light period. Bottom: decreased power in cKO mice of the 0.5–3 Hz delta, 13–30 Hz beta and 30–45 Hz gamma frequency during REM sleep under the light period. Vigilance states classified in **a** and **b** were the same as in Figure 3d. The data recorded at 2000 Hz sampling frequency (six mice each for genotype) were used for the EEG spectrum analysis. *P<0.05, **P<0.01, ***P<0.001 (unpaired *t*-test). Light-colored lines and error bars represent s.e.m. cKO, conditional knockout; EEG, electroencephalography; ILN, intralaminar thalamic nuclei; NREM, non-REM; REM, rapid eye movement.

Abnormal cortical oscillations in schizophrenic patients are well documented.^39^ Multiple studies report consistent abnormalities in theta (4–8 Hz), alpha (8–12 Hz), beta (13–30 Hz) and gamma (30–80 Hz) frequency oscillatory activity in patients with schizophrenia and such abnormal oscillations are proposed to underlie the cognitive symptoms and hallucinations.^40^ We analyzed the EEG data obtained from non-anesthetized mice. The cKO mice showed a significantly reduced power in a wide range of frequencies including the delta, theta, alpha, beta and gamma bands, both during wake and REM sleep and a modest decrease of gamma power during NREM sleep, regardless of the light or dark phase (Figures 4a and b). Consistently, diffuse projections from ILN-Cre-positive cells to cortical areas were observed (Supplementary Figure 4).

### Selective restoration of NMDARs in ILN cells in adult cKO mice rescued the behavioral abnormalities

**Figure 5:**
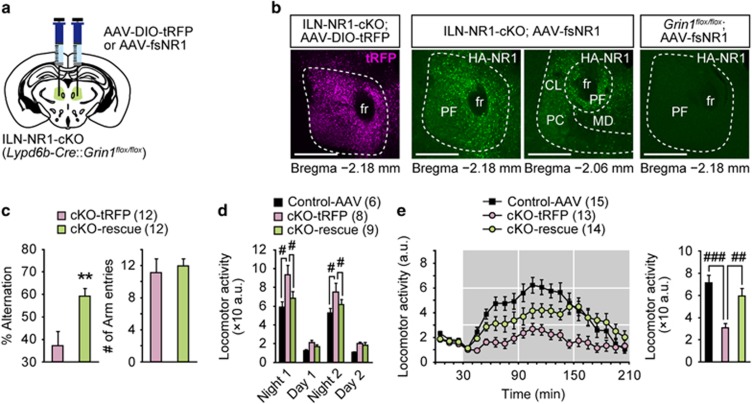
Adult reversal of schizophrenia-related phenotypes by restoration of ILN NMDARs. (**a**) Injection of AAV carrying Cre-dependent NR1 (AAV-fs (floxed-stop) NR1) or control fluorophore (AAV-DIO (double-floxed inversed open reading frame) turboRFP (tRFP)) into the ILN of cKO mice. (**b**) Representative images of immunohistochemistry for tRFP and HA-tag. Expression of tRFP and HA-tagged NR1 was restricted to ILN cells 4 weeks after viral injections in ILN-NR1-cKO; AAV-DIO-tRFP (cKO-tRFP) and ILN-NR1-cKO; AAV-fsNR1 (cKO-rescue) mice. No expression of HA-tagged NR1 was detected in *Grin1*^*flox/flox*^; AAV-fsNR1, because of the lack of Cre. Scale bars, 500 μm. (**c**) Left: in the Y-maze spontaneous alternation test, cKO-rescue mice exhibited a greater percentage of alternations (12 cKO-tRFP and 12 cKO-rescue males, 3 months old). Right: number of arm entries did not significantly differ between genotypes. (**d**) Home cage activity was measured in *Grin1*^*flox/flox*^; AAV-DIO-tRFP or AAV-fsNR1 (control-AAV), cKO-tRFP and cKO-rescue mice. The cKO-rescue mice exhibited moderate locomotor activity (eight cKO-tRFP and nine cKO-rescue males). (**e**) Left: time course of MK-801 induced locomotor activity in control-AAV, cKO-tRFP and cKO-rescue mice. Right: cumulative distance traveled after MK-801 treatment (30–210 min). The cKO-rescue mice exhibited greater responses to MK-801 treatment than cKO-tRFP mice (15 control-AAV (8 females and 7 males), 13 cKO-tRFP (5 females and 8 males) and 14 cKO-rescue (8 females and 6 males), 4 months old). *P<0.01 (unpaired *t*-test). ^#^*P*<0.05, ^##^*P*<0.01, ^###^*P*<0.001 (Tukey’s *post hoc* test). All error bars represent s.e.m. cKO, conditional knockout; HA, hemagglutinin; ILN, intralaminar thalamic nuclei; NMDAR, *N*-methyl-d-aspartate receptor; NREM, non-rapid eye movement.

In cKO mice, NMDAR signaling was likely disrupted from the juvenile stage, suggesting that NMDAR dysfunction during early development is critical for the observed behavioral abnormalities. To evaluate whether restoration of NMDAR function in the adult ILN could effectively ameliorate the behavioral abnormalities, we used a viral rescue strategy. We conditionally re-expressed the NR1 subunit in adult cKO mice using a Cre-dependent AAV vector (Figure 5a). Histologic analysis revealed the restricted expression of hemagglutinin-tagged NR1 or control turboRFP (tRFP) in the ILN (Figure 5b). In the Y-maze spontaneous alternation task, the virally rescued mutant (cKO;AAV-fsNR1 or cKO-rescue) mice exhibited improved performance compared with mutants with control vectors (cKO;AAV-tRFP or cKO-tRFP; Figure 5c). The findings suggest that restoration of NMDAR signaling in the ILN of adult cKO mice was sufficient to rescue the working-memory deficit. We then assessed the home cage locomotor activity (Figure 5d). Although the cKO-tRFP mice exhibited increased activity compared with AAV-transfected *Grin1*^*flox/flox*^ (control-AAV) mice, the increased activity was suppressed in the cKO-rescue mice (Figure 5d). We then tested the virally rescued mutants in the MK-801-induced hyperactivity test (Figure 5e). Although MK-801-induced hyperactivity was largely diminished in the cKO-tRFP mice, the hyperactivity was reversed in the cKO-rescue mice (Figure 5e). The findings suggest that restoration of NMDAR signaling in the adult ILN circuit also reverses various other behavioral deficits and restores MK-801-induced hyperactivity. Importantly, these results confirm that, although some Cre-recombination was detected in other brain areas, the behavioral deficits were primarily due to NMDAR dysfunction in ILN cells.

## Discussion

In the present study, selective disruption of NMDAR signaling in the ILN circuit of mice during developmental stages induced characteristic schizophrenia-like symptoms, some of which were normalized by restoration of NMDAR function in adult mice. These findings contrast with studies showing that NMDAR hypofunction in cortical GABAergic interneurons during early development, but not in adulthood, causes schizophrenia-like phenotypes.^19^ It is likely that NMDAR function in the ILN neurons is required in a post-maturation manner for proper integration of sensory information, while NMDAR function in inhibitory neurons is required for establishing appropriate cortical inhibitory–excitatory neuronal networks during development. Thus, our ILN-NR1-cKO mice provide a valuable platform for future studies of potential post-adolescent treatment of schizophrenia, at least for a subset of schizophrenia.

The inattention and abnormal arousal regulation observed in our model animal support the role of the ILN as a major component of the arousal system.^41^ Previous studies reveal that ILN neurons transfer excitatory inputs from the midbrain reticular formation to cortical areas,^42^ and these ILN circuits might control the transition from relaxed wakefulness to an alert state.^43^ Notably, our model animal shares the physiologic features of human schizophrenia, namely sleep/wake disturbances, including enhanced arousal and decreased NREM sleep. In addition, although the total amount of REM sleep was normal, we observed a shortened REM sleep latency during the light phase. This suggests that the cKO mice indeed have abnormal sleep architecture and the feature resembles that of schizophrenia patients. Thus, dysfunction of the ILN circuit may explain the close relationship between attention deficits and hyperarousal among patients with schizophrenia.^44^ Several studies suggested that memory consolidation occurs during NREM sleep.^45, 46^ It is thus possible that some of the cognitive anomalies resulted from the reduced NREM sleep in the dark phase or from the reduced REM sleep latency during the light phase. In addition to the direct projections from the ILN to the cerebral cortex, indirect regulation of cortical activity via the basal ganglia might also be involved.^27^ Previous studies suggest that ILN-basal ganglia circuits are associated with visual discrimination^26^ and attention,^29^ and that the basal ganglia regulate cortical oscillations^47^ and arousal.^48^ The ILN may act as a hub in the ILN-basal ganglia-cortical circuits. Besides the ILN, the mediodorsal thalamus coordinates thalamo-prefrontal beta-range synchrony, which might also be important for working memory.^22^ Although the ILN and mediodorsal thalamus share a number of anatomic features, individual nuclei preferentially connect with different cortical and subcortical areas,^27^ suggesting differential roles among these thalamic nuclei in regulating cognitive subdomains.

The aberrant cortical oscillations recorded in awake cKO mice may at least in part account for their behavioral defects. Neural oscillations are tightly linked to sensory processing and cognitive function, and alterations of these oscillations is considered a core symptom of schizophrenia.^40^ Theta, alpha, beta and gamma oscillations are associated with a wide range of cognitive functions, including visuospatial attention^49^ and working memory,^50^ and are abnormal in patients with schizophrenia.^51^ Successful cognitive performance in mice is associated with enhanced cortical oscillations.^52^ The abnormal cortical activity in our model animal may interfere with modulation of cortical synchrony according to behavioral demands, similar to the mediodorsal thalamus.^22^ Inhibition of neural activity in the mediodorsal thalamus disrupts thalamo-prefrontal beta-range synchrony, which correlates with impaired working memory.^22^ Similarly, activation of neural activity in thalamic subnuclei, including the ILN, also modulates brain state in behaving animals.^53, 54^ The present findings and those of previous studies suggest that disruption of such thalamic-mediated synchronization mechanisms are responsible for the cognitive deficits observed in our model animal and account for the etiology of schizophrenia.

## Conclusion

The present results support a critical role of the ILN in a broad range of schizophrenia-associated phenotypes, including cognitive-, positive- and hyperarousal-like physiologic symptom domains. The characteristic symptoms of schizophrenia that manifest during adolescence could potentially be ameliorated to some extent by restoring NMDARs or by alternative means in adults. Greater attention should be paid to the ILN when developing therapeutic strategies for treatment-resistant patients with psychiatric disorders.
