# Supplementary material for: Schizophrenia-like phenotypes in mice with NMDA receptor ablation in intralaminar thalamic nucleus cells and gene therapy-based reversal in adults
Source: Transl Psychiatry. 2017 Feb 28;7(2):e1047–. doi: 10.1038/tp.2017.19 (PMC5545645; doi:10.1038/tp.2017.19)
Supplement: Supplementary Information [file tp201719x1.docx]

**Supplementary Information (SI)**

**Schizophrenia-like phenotypes in mice with NMDA receptor ablation in intralaminar thalamic nucleus cells and gene therapy-based reversal in adults**

**By Kosuke Yasuda et al.**

**SI Materials and Methods**

Generation of ILN-specific cKO mice.

PCR for genotyping.

LacZ staining.

Immunohistochemistry.

Electrophysiological experiments *in vitro*.

Behavioral analysis.

Y-maze tests.

Morris water maze.

5-Choice serial reaction-time task.

Open field locomotor activity.

Prepulse-inhibition test.

Home-cage activity and MK-801-induced hyperactivity.

Sleep analysis.

EEG signal analysis.

Virus production and purification.

Data acquisition and analysis.

**SI Text**

Details of statistical analyses

**SI Refferences**

**SI Figures and Legends**

**Supplementary Figure 1.** Representative images of immunohistochemistry in the ILN-Cre; Rosa-NLSLacZ double transgenic mouse.

**Supplementary Figure 2.** Representative images (from 4 mice) of the spatial distribution of Cre recombinase activity in coronal sections from an ILN-Cre::Rosa-NLSLacZ double-transgenic mouse stained with X-gal (blue) and hematoxylin (purple).

**Supplementary Figure 3.** The cKO mice exhibited enhanced arousal during the dark period. For cage change experiments, EEG/EMG recorded mice underwent a cage change at Zeitgeber time (ZT) 2, during the light phase, and after 36 hrs, underwent the second cage change at ZT14, during the dark phase.

**Supplementary Figure 4.** Anatomical characterization of the ILN cells using viral vectors.

**SI Materials and Methods**

**Generation of ILN-specific cKO mice.** Heterozygous ILN-Cre mice were crossed with mice homozygous for the loxP-flanked NR1 allele (*Grin1^flox/flox^*)[^1^](#_ENREF_1) to obtain double heterozygous mice for the Cre transgene and floxed-*Grin1* allele (*Lypd6b-Cre*::*Grin1^flox/^*^+^), and these mice were further crossed with *Grin1^flox/flox^* mice to produce *Lypd6b-Cre*::*Grin1^flox/flox^* mice and littermate control mice, *Grin1^flox/flox^* . The *Lypd6b-Cre*::*Grin1^flox/flox^* mice are referred to as ILN-NR1-cKO mice. For electrophysiologic experiments, ILN-NR1-cKO mice were crossed with *Grin1^flox/flox^*::*Gt(ROSA)26Sort^m3(CAG-EYFP)Hze^*^[2](#_ENREF_2)^ to obtain *Lypd6b-Cre*:: *Grin1^flox/flox^*:: *Gt(ROSA)26Sort^m3(CAG-EYFP)Hze^* (cKO::Rosa26-LSL-EYFP) mice, and the ILN-Cre mice were crossed with *Gt(ROSA)26Sort^m3(CAG-EYFP)Hze^* mice to obtain ILN-Cre:: *Gt(ROSA)26Sort^m3(CAG-EYFP)Hze^* (ILN-Cre::Rosa26-LSL- EYFP) control mice.

**PCR for genotyping.** We used PCR to determine the mouse genotype, using the following primers: *Cre* forward, 5’- ACC TGA TGG ACA TGT TCA GGG ATC G -3’ and *Cre* reverse, 5’- TCC GGT TAT TCA ACT TGC ACC ATG C -3’, producing a 108-bp fragment from the Cre allele; *Grin1* forward (a), 5’- TGT GCT GGG TGT GAG GGT TG -3’ and *Grin1* reverse (b), 5’- GTG AGC TGC ACT TCC AGA AG -3’, producing 220-bp and 280-bp fragments from wild-type and knock-in *Grin1* alleles, and *CAG-EFYP* forward (a), 5’- AAG GGA GCT GCA GTG GAG TA -3’, *CAG-EYFP* reverse (a), 5’- CCG AAA ATC TGT GGG AAG TC -3’, *CAG-EFYP* forward (b), 5’- GGC ATT AAA GCA GCG TAT CC -3’, and *CAG-EYFP* reverse (b), 5’- ACA TGG TCC TGC TGG AGT TC -3’, producing 297-bp and 212-bp fragments from wild-type and knock-in *EYFP* alleles, respectively.

**LacZ staining.** Mice were transcardially perfused with 4% paraformaldehyde in 0.1 M sodium phosphate buffer, and post-fixed by the same fixative for 3 h. Brain sections (50-μm thick) were prepared on a Vibratome (Pro7; Dosaka, Kyoto, Japan) and collected in phosphate-buffered saline (PBS). After rinsing in PBS, the sections were incubated at 37°C overnight in X-gal staining solution (0.1 M PB [pH 7.5], 20 mM Tris–HCl [pH 7.5], 5 mM K_3_[Fe(CN)_6_], 5 mM K_4_[Fe(CN)_6_], 2 mM MgCl_2_, 1 mg/ml X-gal).

**Immunohistochemistry.** Mice were transcardially perfused with 4% paraformaldehyde in 0.1 M sodium phosphate buffer and post-fixed in the same fixative overnight. Brain sections were prepared on a Vibratome (Pro7, DOSAKA) and collected in PBS. Free-floating sections were first treated with TBST (100 mM Tris-HCl, 140 mM NaCl, and 0.2% Triton-X), then with 0.8% Block Ace (DS Pharma Biomedical Co. Ltd) for 1 h. Vibratome sections (50-μm thick) were incubated with primary antibody diluted in 0.4% Block Ace (Bio-Rad) at 4°C overnight (mouse anti-NeuN, 1/100, Chemicon, NAB337; rabbit anti-β-galactosidase, 1/10000, Promega, Z3781; rabbit anti-NR1, 1/200, Frontier Institute, GluRz1-Rb-Af720; mouse anti-HA, 1/500, ABM, G036), and then incubated with secondary antibodies diluted in 0.4% Block Ace for 1 h at room temperature (Alexa 488-conjugated anti-mouse IgG, 1/2000, Molecular Probes, A-21121; Alexa 546-conjugated anti-rabbit IgG, 1/2000, Molecular Probes, A-11010; Alexa 594-conjugated anti-mouse IgG, 1/2000, Molecular Probes, A-11032). After rinsing with PBST, the sections were mounted on glass slides. Images were obtained with a confocal microscope (FluoView FV1000, Olympus).

**Quantitative RT-PCR.** Circular tissue punches (1-mm diameter) were obtained from 140-μm thick frozen coronal brain sections from 2-month-old mice (3 females/group) and stored at −80°C until assayed. Circular tissue punches was lysed with QIAzol Lysis Reagent (QIAGEN, 79306), and total RNA was extracted and purified with an miRNeasy Mini Kit (QIAGEN, 217004) following the manufacturer’s instructions. The mRNA levels of individual genes were analyzed using a SuperScript III Two-Step qRT-PCR kit (Invitrogen, 11734-050) and SYBR Premix Ex Taq II (Takara, RR081A). PCR was performed in a 7500 Real-Time PCR System (Applied Biosystems). The amount of mRNA of the target genes, normalized to that of an endogenous control (*glyceraldehyde-3-phosphate dehydrogenase*, *Gapdh*), was calculated with a standard curve. The standard curve was generated using a 4-log spanning serial dilution of cDNA samples derived from circular tissue punches of wild-type mice. The following primers were used: *Grin1* forward (b), 5’- TAC AAG CGA CAC AAG GAT GC -3’ and *Grin1* reverse (b), 5’- TCA GTG GGA TGG TAC TGC TG -3’, producing 98-bp fragments; *Gapdh* forward, 5’- GGG TTC CTA TAA ATA CGG ACT GC -3’ and *Gapdh* reverse, 5’- CCA TTT TGT CTA CGG GAC GA -3’, producing 112-bp fragments.

**Electrophysiological experiments *in vitro*.** ILN-Cre; Rosa26-EYFP mice and ILN-Cre; NR1 flox/flox; Rosa26-EYFP mice were used for the control and cKO experiments, respectively, at postnatal day 27–30. The brains were removed under deep isoflurane anesthesia. Acute parasagittal slices (300-μm thick) were prepared using a vibratome (Leica VT1200 S, Leica Microsystems) in ice-cold artificial cerebrospinal fluid (ACSF) containing (in mM): 126 NaCl, 3 KCl, 1.25 NaH_2_PO_4_, 2 CaCl_2_, 1 MgCl_2_, 26 NaHCO_3_, and 10 glucose; and bubbled with 95% O_2_ and 5% CO_2_. The slices were recovered in a submerged-type chamber filled with ACSF at 32°C for 30 min, and then maintained at room temperature for at least 30 min before recording.

During recording, the slices were perfused with ACSF at 26°C to 30°C. Infrared differential interference contrast video microscopy (BX-51, Olympus) with a 40×, 0.8 NA water immersion lens was used to visualize the neurons. Patch pipettes (3–8 MΩ) were filled with an intracellular solution containing (in mM): 130 potassium gluconate, 8 KCl, 1 MgCl_2_, 0.6 EGTA, 10 HEPES, 3 Na_2_ATP, 0.5 Na_2_GTP, 10 Tris-phosphocreatine, and 0.2% biocytin (pH adjusted to 7.35 with KOH). Whole-cell patch-clamp recordings were performed on EYFP(+) neurons just anterior to the fasciculus retroflexus. Current signals were recorded using a MultiClamp 700B amplifier (Molecular Devices), low-pass filtered at 10 kHz, digitized at 20 kHz, and stored using pClamp10 (Molecular Devices). Data were analyzed using MATLAB (Mathworks). Membrane potentials are given with a correction for the liquid junction potential of −13 mV. Series resistance compensation of up to 50% was used for some recordings.

A glass stimulating electrode was placed at the slice surface above the recorded cell. Excitatory postsynaptic currents (EPSCs) were evoked by electrical stimulation of 100-μs duration at 0.2 Hz in the presence of 10 or 50 μM bicuculline methiodide (a GABA_A_ receptor antagonist; Tocris Bioscience) and 10 μM strychnine hydrochloride (a glycine receptor antagonist; Abcam). EPSCs were recorded at the holding potentials of −70 mV, 0 mV, and +40 mV. Stimulation was typically repeated 20 to 50 times at each holding potential. Cells for which stimulation elicited outward inhibitory postsynaptic currents at a holding potential of 0 mV were discarded from the analyses. The decay time constant of the EPSCs was calculated by fitting between 10% and 90% of the current peak and baseline. A decay time constant of 8 ms was used to determine the presence of NMDA currents (Figure 1g). For blocking experiments, 10 μM 2,3-Dioxo-6-nitro-1,2,3,4-tetrahydrobenzo[f]quinoxaline-7-sulfonamide disodium salt (NBQX, an AMPA receptor blocker; Tocris Bioscience) and 50 μM D-(-)-2-Amino-5-phosphonopentanoic acid (APV, an NMDA receptor blocker; Tocris Bioscience) were added to the ACSF while the cells were held at +40 mV and stimulation was applied at 0.2 Hz. spontaneous EPSCs (sEPSCs) were recorded at the holding potentials of −70 mV. To construct cumulative probability distributions of sEPSC amplitude, 75 random events were selected in individual cells using MATLAB and events from control, cKO_n+ and cKO_n− cells were respectively pooled. Statistical comparisons of cumulative distributions were made using the Kolmogorov-Smirnov test. Biocytin-filled neurons were visualized using streptavidin-Cy5 (GE Healthcare).

**Behavioral analysis.** All behavioral tasks were performed, as previously described,[^3^](#_ENREF_3)^,^ [^4^](#_ENREF_4) during the light phase. The first cohort of mice was tested in the Morris water maze. The second cohort of mice was tested in the open field, home cage activity, 5-choice serial reaction time, prepulse inhibition, and MK-801 induced hyperactivity tests. The third cohort of mice was tested in the Y-maze and three-chamber social interaction test. The fourth cohort of mice was microinjected with adeno-associated virus vectors and tested in the Y-maze test. The fifth cohort of mice was microinjected with adeno-associated virus vectors and tested in the home cage activity and MK-801 induced hyperactivity tests.

**Y-maze tests.** A Y maze with three identical arms made of transparent Plexiglass (39.5 × 3 × 12 cm) 120° apart was placed in the center of a diffusely illuminated room (30 lux). Each mouse was placed at the end of one arm facing the center and allowed to freely explore the apparatus with the experimenter out of sight. All sessions were video-recorded through a camera mounted above the maze and evaluated using EthoVision XT 8.0 (Noldus). Entry into each arm was scored for 5 min beginning from the first entry, as described previously.[^4^](#_ENREF_4) Alternation behavior was defined as consecutive entries into each of the three arms without repetition (i.e., ABC, BCA, CAB, ACB, CBA, or BAC). We defined the percentage of spontaneous alternations as the actual alternations divided by the possible alternations (total arm entries – 2) × 100.

**Morris water maze.** The Morris water maze test was performed as previously described with some modifications.[^3^](#_ENREF_3) The water pool used was 150 cm in diameter. The temperature of the water was held constant at 25 ± 1°C. Mice were given 4 trials per day for 9 d in the hidden platform task. During the acquisition period, four different starting points were used and the starting positions were varied pseudo-randomly over the trials. A probe test was performed on the final day of the acquisition session. In the probe test, the platform was removed from the maze and each mouse was allowed to swim for 90 s. The time spent in the target or pseudo target zones (30 cm radius centered at the platform or pseudo platform positions) in each quadrant of the maze was recorded.[^5^](#_ENREF_5) The visible platform test was performed for 4 d. In the visible platform task, a black cubic landmark was attached to the platform. Mouse movement in the water maze was recorded by a camera and analyzed using software (O’Hara & Co.).

**5-Choice serial reaction-time task.** The 5-CSRTT was performed as previously described, with some modifications.[^3^](#_ENREF_3) Male and female mice were food-restricted to reduce their body weight to approximately 85% of their free-feeding weight. After the mice were trained to consume food pellets delivered automatically at 45-s intervals from a pellet dispenser for 15 min daily for 3 d (Food training, aperture), the mice were trained to associate nose-pokes with food delivery for 30 min during the next 10 d (Nose-poke training, 100 trials/d). In the Nose-poke training, the stimulus duration of green signal lights behind all of the holes was 30 s. In the following sessions (spatial stage 1–8), the mice were trained to respond to presentations of a green signal light that was pseudo-randomly displayed in one of five apertures. Each trial commenced with an inter-trial interval, during which any response to a hole was recorded as a premature response. The stimulus was then displayed, followed by a limited holding period. In spatial stages 2 through 8, when a mouse did not perform a nose-poke within the limited holding period (omission) or performed a nose-poke into a wrong hole (incorrect response), the house light was extinguished for 2 s. When a mouse continued nose-pokes in any hole after a correct response, but before collecting food pellets, the responses were recorded as perseverative responses. The mean latency of the mice to make correct and incorrect responses was calculated as a measure of its processing speed in the task.

The inter-trial interval, stimulus duration, and limited holding periods were 2 s, 30 s, and 30 s (spatial stage 1, 14 sessions); 10 s, 30 s, and 30 s (spatial stage 2, 5 sessions); 10 s, 10 s, and 10 s (spatial stage 3, 3 sessions); 15 s, 10 s, and 10 s (spatial stage 4, 3 sessions); 15 s, 5 s, and 5 s (spatial stage 5, 5 sessions); 15 s, 2 s, and 2 s (spatial stage 6, 5 sessions); 15 s, 1 s, and 2 s (spatial stage 7, 2 sessions); and 15 s, 0.6 s, and 2s (spatial stage 8, 2 sessions), respectively. Mice were required to complete 100 trials within a 30-min session. When an animal reached a performance criterion (completed 100 trials, > 80% correct response, < 20% omissions for 3 of 5 consecutive days) at spatial stage 5, it was then tested in the following spatial stages in which the stimulus duration was reduced to 2.0 s (stage 6), 1.0 s (stage 7), and 0.6 s (stage 8).

**Open field locomotor activity.** Each mouse was placed in the center of an open field (50 × 50 × 50 cm, 70 lux) and allowed to freely explore the apparatus for 10 min. The activity of the mice was recorded by a camera and analyzed using software (O’Hara & Co.) to assess the total distance moved (cm), time in the center (s), and rearing activity (count).

**Prepulse-inhibition test.** Prepulse inhibition (PPI) testing was conducted in four startle chambers (O’Hara & Co.) as described previously, with some modifications[^3^](#_ENREF_3). The test comprised 45 trials. Nine different trial types were presented: non-stimulus trials, trials with an acoustic stimulus (40-ms white noise, either 80, 90, 100, 110, or 120 dB) alone, and trials in which the prepulse stimulus (20-ms white noise, either 70, 75, or 80 dB) had an onset 100 ms before the onset of the acoustic startle stimulus (40-ms white noise, 120 dB). The different trial types were presented in blocks of nine, in randomized order in each block, with a mean inter-trial interval of 15 s (range: 10–20 s). The percent startle response was recorded by measuring the amplitude of the startle reflex in response to the prepulse stimulus and the startle stimulus, and calculated as 100 − [response amplitude of the prepulse stimulus + response amplitude of the startle stimulus)/response amplitude of the startle stimulus] at each prepulse sound level (70, 75, and 80 dB).

**Home-cage activity and MK-801-induced hyperactivity.** To measure locomotor activity in the home cage, each mouse was placed in a transparent cage (20.7 [W] × 36.5 [L] × 14 [H]) containing bedding material, and horizontal and vertical activities were monitored continuously for 2 d after 1-d habituation using infrared area sensors **(**SCANET Behavioral Analysis System; Melquest), as described previously.[^3^](#_ENREF_3) To examine MK-801 induced hyperactivity, the mice were injected subcutaneously with a noncompetitive NMDAR antagonist, MK-801 ((+)-MK 801 maleate [TOCRIS], 0.3 mg per kg in 0.9% saline), or solvent (0.9% saline). The mice were tested in a home cage placed in the SCANET Behavioral Analysis System (Melquest). The activity of the mice was measured for 240 min through infrared beam breaks and analyzed every 1 min. The session began with a 30-min habituation period, and mice were subsequently injected with MK-801 or solvent by an experimenter.

**Sleep analysis.** Sleep analysis was carried out according to the procedure described previously.[^6^](#_ENREF_6) First, operations were performed under aseptic conditions. Mice were anesthetized with isoflurane and fixed in a stereotaxic apparatus (Leica Angle Two). The body temperature of the animals was maintained at 37°C using a heating pad (TR-200, FST). The mice were implanted with electroencephalography (EEG) and electromyography (EMG) electrodes. EEG electrodes were stainless steel screws implanted epidually over the bilateral frontal areas and unilateral parietal area. EMG electrodes were stainless steel Teflon-coated wires placed bilaterally into the trapezius muscle. For reducing sensitization to pain, 2% lidocaine-gel was applied before and after the surgery. After surgery, the mice were fully recovered from anesthesia in a warm environment before being returned to the holding room. The health condition of the operated mice were observed daily. The mice were allowed to recover for at least 2 weeks in their home cages before EEG recording. EEG/EMG signals were recorded using a preamplifier connected to a data acquisition system (8200-K1-SE, Pinnacle Technology) and Sirenia Software (Pinnacle Technology). For sleep/wake cycle analysis, EEG/EMG signals were recorded at 2-kHz or 200 Hz sampling frequency for 48 h. For cage change experiments, EEG/EMG recorded mice underwent a cage change at Zeitgeber time (ZT) 2, during the light phase, and after 36 hrs, underwent the second cage change at ZT14, during the dark phase. After the second cage change, EEG/EMG signals were recorded for an additional 8 hrs. EEG signals were subjected to fast Fourier transform and further analysis using SleepSign (Kissei Comtec). The vigilance state in each 10-s epoch was manually classified as REM sleep, NREM sleep, or wakefulness, based on absolute delta (0.5–4 Hz) power, theta (6–10 Hz) power to delta power ratio, and the integral of EMG signals. If a single epoch contained multiple states, the state with the highest occupancy was assigned. The vigilance state scoring was conducted by an experimenter completely blind to the genotypes. REM sleep latency was defined based on a previous study.[^7^](#_ENREF_7) REM latency was defined as the time elapsing from sleep onset to the beginning of first episode of REM sleep in the cage change experiments.

**EEG signal analysis.** EEG signals recorded at 2-kHz sampling frequency were low-pass filtered (<200 Hz) and converted into power spectra by fast Fourier transform every sleep/wake epochs. The power spectra were smoothed by low-pass filtering and spline interpolation. The flattened spectra were normalized by subtraction of averaged power at the frequency range from 150 to 200 Hz, where the power components are supposed to common noise at the range. The normalized spectra were averaged over subjects and recording days in each light/dark cycle and sleep/wake condition. Epochs representing the transision form one state to the other were excluded from the analysis. The grouped power spectra were averaged at frequency ranges divided into six as follows: 0.5–3, 4–8, 8–12, 13–30, 30–45, and 55–80 Hz. The analysis was performed using custom software written in MATLAB (Mathworks).

**Virus production and purification.** The turboRFP-coding sequences were amplified by PCR along with *Asc*I and *Nhe*I linkers from a plasmid, TtTMPV-Neo (shRen) (Addgene, Plasmid #27993), using the following primers: *turboRFP* forward: 5’- GCT AGC TAG CCA CCA TGA GCG AGC TGA TCA AGG AG -3’, *turboRFP* reverse: 5’- TTG GCG CGC CTC ATC TGT GCC CCA GTT TGC -3’. PCR was performed in a final volume of 100 μl using 5 μl of each primer (10 μM), 20 μl of 5× Phusion HF buffer (New England Biolabs, M0531S), 8 μl of 2.5 mM dNTP, 59 μl H_2_O, and 1 μl of the template DNA. The PCR products were digested with AscI and NheI. The fragments containing turboRFP were ligated in the antisense direction into *Asc*I and *Nhe*I sites of pAAV-EF1a-DIO-EYFP-WPRE-HGHpA (Addgene, Plasmid #20296), to yield the plasmid pAAV-DIO-turboRFP.

Viruses were produced using a triple-transfection, helper-free method and purified using an ultracentrifugation protocol, with some modifications.[^8^](#_ENREF_8) The 293FT cells (Invitrogen) were cultured in 30 ml complete medium (Dulbecco’s modified Eagle’s medium containing 10% heat-inactivated fetal bovine serum, 1 mM sodium pyruvate solution, 0.075% sodium bicarbonate solution, and 1% penicillin-streptomycin-L-glutamine solution) in 225 cm^2^ cell culture flasks (Nunc, 159934) at 37°C/5% CO_2_, until 293FT cells reached approximately 70% to 80% confluence. They were then transfected with pHelper, pAAV-DJ/8 (Cell Biolabs), and pAAV-DIO-turboRFP or pAAV-fsNR1 (provided by Dr. Richard D. Palmiter of the University of Washington).[^9^](#_ENREF_9) Plasmids (20 μg each) were mixed with 293fectin™ transfection reagent (Invitrogen) in 300 μl Opti-MEM (Invitrogen, 11058021) and incubated for 30 min at 25°C before the mixture was transferred to the 70% to 80% confluent 293FT cells. After 3 d incubation at 37°C/5% CO_2_, each lysate was collected into 50-ml conical tubes and spun down by centrifugation for 30 min at 2500 rpm, followed by filtration. Each cleared lysate was moved into ultracentrifuge tubes and 20% sucrose/PBS solution was added to the bottom of the tubes before centrifuging in a Beckman SW-28 rotor at 22,000 rpm at 4 ºC for 2 h. After pouring out the supernatant and drying the sides of each tube with a Kimwipe, viruses in the pellet at the bottom were extracted in 100 µl of cold PBS by gently pipetting the PBS up and down. The final purified viruses were stored at −80°C. Genomic AAV titers were determined by quantitative real-time PCR using a modification of a published protocol.[^10^](#_ENREF_10) The following primers were used: *Eif1a* forward: 5’- GAG TTT CCC CAC ACT GAG TG -3’, *Eif1a* reverse: 5’- GAG GCT TGA GAA TGA ACC AAG A -3’. A fragment length of 201 base pairs of the quantitative PCR product was expected using the primers.

**Data acquisition and analysis.** Behavioral tests and sleep analysis were performed by an investigator under strict genotype-blind conditions, and *in vitro* electrophysiologic data acquisition was performed by an investigator with knowledge of the identity of the experimental group. No statistical methods were used to predetermine sample sizes, but the sample sizes were similar to those reported previously. No randomization was used to assign experimental groups. No single data points were excluded. Viral-injected animals in which the injections missed the intended target were excluded.

**SI Text**

**Details of statistical analyses**

**Figure 1d.** *n* = 6 samples (3 females) for each group (2 months-old). There was significant difference between genotype (Wilcoxon rank sum test, *E*(*U*) = 18, *V*(*U*) = 38.0, *z* = 2.88, *P* = 0.00395).

**Figure 1j.** Control, *n* = 33 cells; cKO_n+ = 20 cells; cKO_n− = 36 cells. For cumulative probability of EPSC amplitude, there was a significant difference between groups (Kolmogorov-Smirnov test, *P* < 0.001). For amplitude of EPSCs, there was a significant effect of genotype (Kruskal-Wallis test, *χ*^2^(2, 86) = 12.2, *P* = 0.00220). Amplitude of EPSCs in cKO_n− was significantly different compared to control (Steel-Dwass multiple comparison test, *T* = 3.38, *P* = 0.00210). Amplitude of EPSCs in cKO_n− was not significantly different compared to cKO_n+ (Steel-Dwass multiple comparison test, *T* = 1.76, *P* = 0.180). Amplitude of EPSCs in cKO_n+ was not significantly different compared to control (Steel-Dwass multiple comparison test, *T* = 1.56, *P* = 0.260). For frequency of EPSCs, there was a significant effect of genotype (Kruskal-Wallis test, *χ*^2^(2, 86) = 15.0, *P* = 0.000562). Frequency of EPSCs in cKO_n− was significantly different compared to control (Steel-Dwass multiple comparison test, *T* = 3.11, *P* = 0.00531). Frequency of EPSCs in cKO_n− was not significantly different compared to cKO_n+ (Steel-Dwass multiple comparison test, *T* = 0.872, *P* = 0.655). Frequency of EPSCs in cKO_n+ was significantly different compared to control (Steel-Dwass multiple comparison test, *T* = 3.41, *P* = 0.00183).

**Figure 2a. Left**, *n* = 10 and 11 males for each groups (2.5 months-old). These was significant difference between genotypes (unpaired *t*-test, *t*(19) = 2.15, *P* = 0.0450).

**Figure 2a. Right**, *n* = 10 and 11 males for each groups (2.5 months-old). These was no significant difference between genotypes (unpaired *t*-test, *t*(19) = 1.584, *P* = 0.1297).

**Figure 2b. Left**. *n* = 7 and 9 males for each groups (6.5 months-old). There was significant genotype × day interaction (mixed between-within subjects ANOVA, genotype × day interaction, *F*(8, 112) = 3.18, *P* = 0.00271). Therefore, simple main effects of genotype at each time point was tested, and significant simple main effects was detected at the following time points (one-way ANOVA, at 7 d, *F*(1, 36) = 5.98, *P* = 0.0194; at 9 d, *F*(1, 36) = 5.58, *P* = 0.0237).

**Figure 2b. Center.** *n* = 7 and 9 males for each groups (6.5 months-old). There was significant genotype × zone interaction (mixed between-within subjects ANOVA, genotype × zone interaction, *F*(3, 42) = 5.18, *P* = 0.00390) Therefore, simple main effects of genotype at each time point was tested, and significant simple main effect was detected at the target zone (one-way ANOVA, *F*(1, 46) = 15.2, *P* = 0.000256).

**Figure 2b. Right.** *n* = 7 and 9 males for each groups (6.5 months-old). There was no significant interaction between genotype and day nor a significant main effect of genotype (mixed between-within subjects ANOVA, main effects of genotype, *F*(1, 14) = 2.84, *P* = 0.114, genotype × day interaction, *F*(3, 40) = 1.64, *P* = 0.194).

**Figure 2d.** *n* = 22 control (13 females and 9 males) and 18 mutant (9 females and 9 males) mice (8 months-old). Data from females and males were pooled, as neither main effects of sex nor interaction effects between sex and genotype were detected (mixed between-within subjects ANOVA, main effects of sex, *F*(1, 36) = 1.76, *P* = 0.193, sex × genotype interaction, *F*(1, 36) = 0.169, *P* = 0.683). There was no significant interaction between genotype and session nor a significant main effect of genotype (mixed between-within subjects ANOVA, main effects of genotype, *F*(1, 38) = 0.0196, *P* = 0.889, genotype × session interaction, *F*(13, 494) = 1.27, *P* = 0.225).

**Figure 2e. Left.** *n* = 22 control (13 females and 9 males) and 18 mutant (9 females and 9 males) mice (8 months-old). Data from females and males were pooled, as neither main effects of sex nor interaction effects between sex and genotype were detected (mixed between-within subjects ANOVA, main effects of sex, *F*(1, 36) = 0.0191, *P* = 0.891, sex × genotype interaction, *F*(1, 36) = 0.317, *P* = 0.577). There was no significant interaction between genotype and stimulus duration nor a significant main effect of genotype (mixed between-within subjects ANOVA, main effects of genotype, *F*(1, 38) = 1.20, *P* = 0.280, genotype × stimulus duration interaction, *F*(3, 114) = 0.290, *P* = 0.832).

**Figure 2e. Right.** *n* = 22 control (13 females and 9 males) and 18 mutant (9 females and 9 males) mice (8 months-old). Data from females and males were pooled, as neither main effects of sex nor interaction effects between sex and genotype were detected (mixed between-within subjects ANOVA, main effects of sex, *F*(1, 36) = 0.455, *P* = 0.504, sex × genotype interaction, *F*(1, 36) = 0.475, *P* = 0.495). There was no significant interaction between genotype and stimulus duration nor a significant main effect of genotype (mixed between-within subjects ANOVA, main effects of genotype, *F*(1, 38) = 0.289, *P* = 0.594, genotype × stimulus duration interaction, *F*(3, 114) = 0.226, *P* = 0.878).

**Figure 2f.** *n* = 22 control (13 females and 9 males) and 18 mutant (9 females and 9 males) mice (8 months-old). Data from females and males were pooled, as neither main effects of sex nor interaction effects between sex and genotype were detected (mixed between-within subjects ANOVA, main effects of sex, *F*(1, 36) = 0.139, *P* = 0.711, sex × genotype interaction, *F*(1, 36) = 1.12, *P* = 0.297). There was no significant interaction between genotype and stimulus duration, and there was a significant main effect of genotype (mixed between-within subjects ANOVA, main effects of genotype, *F*(1, 38) = 7.50, *P* = 0.00933, genotype × stimulus duration interaction, *F*(3, 114) = 1.02, *P* = 0.387).

**Figure 2g.** *n* = 22 control (13 females and 9 males) and 18 mutant (9 females and 9 males) mice (8 months-old). Data from females and males were pooled, as neither main effects of sex nor interaction effects between sex and genotype were detected (mixed between-within subjects ANOVA, main effects of sex, *F*(1, 36) = 1.65, *P* = 0.208, sex × genotype interaction, *F*(1, 36) = 0.143, *P* = 0.708). There was no significant interaction between genotype and stimulus duration, and there was a significant main effect of genotype (mixed between-within subjects ANOVA, main effects of genotype, *F*(1, 38) = 7.19, *P* = 0.0108, genotype × stimulus duration interaction, *F*(3, 114) = 2.53, *P* = 0.0610).

**Figure 2h.** *n* = 22 control (13 females and 9 males) and 18 mutant (9 females and 9 males) mice (8 months-old). Data from females and males were pooled, as neither main effects of sex nor interaction effects between sex and genotype were detected (mixed between-within subjects ANOVA, main effects of sex, *F*(1, 36) = 0.453, *P* = 0.505, sex × genotype interaction, *F*(1, 36) = 0.143, *P* = 0.708). There was no significant interaction between genotype and stimulus duration, and there was a significant main effect of genotype (mixed between-within subjects ANOVA, main effects of genotype, *F*(1, 38) = 4.268, *P* = 0.0457, genotype × stimulus duration interaction, *F*(3, 114) = 0.999, *P* = 0.396).

**Figure 2i.** *n* = 22 control (13 females and 9 males) and 18 mutant (9 females and 9 males) mice (8 months-old). Data from females and males were pooled, as neither main effects of sex nor interaction effects between sex and genotype were detected (mixed between-within subjects ANOVA, main effects of sex, *F*(1, 36) = 0.0566, *P* = 0.813, sex × genotype interaction, *F*(1, 36) = 0.566, *P* = 0.457). There was no significant interaction between genotype and stimulus duration nor a significant main effect of genotype (mixed between-within subjects ANOVA, main effects of genotype, *F*(1, 38) = 3.61, *P* = 0.0651, genotype × stimulus duration interaction, *F*(3, 114) = 1.85, *P* = 0.141).

**Figure 3a. Left.** *n* = 16 control (8 females and 8 males) and 17 mutant (8 females and 9 males) mice (3 months-old). There was no significant difference between groups (unpaired *t*-test, *t*(24.8) = 1.63, *P* = 0.116).

**Figure 3a. Right.** *n* = 16 control (8 females and 8 males) and 17 mutant (8 females and 9 males) mice (3 months-old). There was significant difference between groups (unpaired *t*-test, *t*(31) = 2.90, *P* = 0.00678).

**Figure 3b. Left.** *n* = 8 and 9 females for each group (3.5 months-old). There was significant genotype × time interaction (mixed between-within subjects ANOVA, genotype × time interaction, *F*(47, 705) = 1.85, *P* = 0.000627, main effects of genotype, *F*(1, 15) = 3.45, *P* = 0.0829). Therefore, simple main effects of genotype at each time point was tested, and significant simple main effects was detected at the following time points (one-way ANOVA, at 21:00 day1, *F*(1, 92) = 6.91, *P* = 0.0101; at 22:00 day1, *F*(1, 92) = 4.56, *P* = 0.0354; at 7:00 day1, *F*(1, 92) = 18.3, *P* < 0.001; at 22:00 day2, *F*(1, 92) = 4.45, *P* = 0.0366; at 23:00 day2, *F*(1, 92) = 4.53, *P* = 0.0359; at 24:00 day2, *F*(1, 92) = 5.13, *P* = 0.259; at 3:00 day2, *F*(1, 92) = 4.79, *P* = 0.0312, at 6:00 day2, *F*(1, 92) = 6.15, *P* = 0.149; at 7:00 day2, *F*(1, 92) = 22.1, *P* < 0.001).

**Figure 3b. Right.** There was no significant interaction between genotype and time nor a significant main effect of genotype (mixed between-within subjects ANOVA, main effects of genotype, *F*(1, 15) = 3.45, *P* = 0.0829, genotype × time interaction, *F*(3, 45) = 2.35, *P* = 0.0852).

**Figure 3c. Left.** *n* = 14 controls (6 females and 8 males) and 13 mutants (6 females and 7 males) at the age of 12 months. Data from females and males were pooled, as neither main effects of sex nor interaction effects between sex and genotype were detected (mixed between-within subjects ANOVA, main effects of sex, *F*(1, 23) = 0.00133, *P* = 0.971, sex × genotype interaction, *F*(1, 23) = 0.00602, *P* = 0.939). There was no significant interaction between genotype and prepulse, and there was significant difference between genotype (mixed between-within subjects ANOVA, main effects of genotype, *F*(1, 25) = 12.7, *P* = 0.00149, genotype × prepulse interaction, *F*(2, 50) = 0.132, *P* = 0.877).

**Figure 3c. Right.** *n* = 14 controls (6 females and 8 males) and 13 mutants (6 females and 7 males) at the age of 12 months. Data from females and males were pooled, as neither main effects of sex nor interaction effects between sex and genotype were detected (mixed between-within subjects ANOVA, main effects of sex, *F*(1, 23) = 2.88, *P* = 0.103, sex × genotype interaction, *F*(1, 23) = 0.0425, *P* = 0.839). There was no significant interaction between genotype and pulse nor a significant main effect of genotype (mixed between-within subjects ANOVA, main effects of genotype, *F*(1, 25) = 0.649, *P* = 0.428, genotype × pulse interaction, *F*(2, 50) = 0.513, *P* = 0.602).

**Figure 3d.** *n* = 8 control and 8 mutant males (4–6 months-old). For wake, simple main effect of genotype at each time point was tested, and a significant effect was detected at the following time points (one-way ANOVA: (at ZT10) *F*(1, 14) = 5.446, *P* = 0.035, (at ZT12) *F*(1, 14) = 5.535, *P* = 0.034, (at ZT13) *F*(1, 14) = 6.953, *P* = 0.020, (at ZT14) *F*(1, 14) = 5.734, *P* = 0.031, (at ZT15) *F*(1, 14) = 5.364, *P* = 0.036)). For NREM, simple main effect of genotype at each time point was tested, and a significant effect was detected at the following time points (one-way ANOVA: (at ZT11) *F*(1, 14) = 4.716, *P* = 0.048, (at ZT12) *F*(1, 14) = 5.398, *P* = 0.036, (at ZT13) *F*(1, 14) = 7.297, *P* = 0.017, (at ZT14) *F*(1, 14) = 6.064, *P* = 0.027), (at ZT15) *F*(1, 14) = 5.925, *P* = 0.029)). For REM, simple main effect of genotype at each time point was tested, and a significant effect was detected at the following time points (one-way ANOVA: (at ZT10) *F*(1, 14) = 5.730, *P* = 0.031).

**Figure 3e.** *n* = 8 control and 8 mutant males (4–6 months-old). For wake during the whole day, there was significant difference between genotype (unpaired *t*-test, *t*(14) = 2.82, *P* = 0.0137). For NREM during the whole day, there was significant difference between genotype (unpaired *t*-test, *t*(14) = 2.79, *P* = 0.0144). For REM during the whole day, there was no significant difference between genotype (unpaired *t*-test, *t*(14) = 1.02, *P* = 0.325).

**Figure 3f.** *n* = 8 control and 8 mutant males (4–6 months-old). For the number of wake episodes during the light period, there was no significant difference between genotype (unpaired *t*-test, *t*(14) = 0.791, *P* = 0.442). For the number of NREM episodes during the light period, there was no significant difference between genotype (unpaired *t*-test, *t*(14) = 1.019, *P* = 0.325). For the number of REM episodes during the light period, there was no significant difference between genotype (unpaired *t*-test, *t*(14) = 1.113, *P* = 0.285). For the number of wake episodes during the dark period, there was a significant difference between genotype (unpaired *t*-test, *t*(14) = 3.841, *P* = 0.002). For the number of NREM episodes during the dark period, there was a significant difference between genotype (unpaired *t*-test, *t*(14) = 4.203, *P* = 0.001). For the number of REM episodes during the dark period, there was no significant difference between genotype (unpaired *t*-test, *t*(9.733) = 0.791, *P* = 0.448).

**Figure 3g.** *n* = 8 control and 8 mutant males (4–6 months-old). For the duration of wake episodes during the light period, there was no significant difference between genotype (unpaired *t*-test, *t*(14) = 1.879, *P* = 0.081). For the duration of NREM episodes during the light period, there was no significant difference between genotype (unpaired *t*-test, *t*(14) = 0.508, *P* = 0.619). For the duration of REM episodes during the light period, there was no significant difference between genotype (unpaired *t*-test, *t*(14) = 0.393, *P* = 0.700). For the duration of wake episodes during the dark period, there was a significant difference between genotype (unpaired *t*-test, *t*(9.802) = 4.382, *P* = 0.001). For the duration of NREM episodes during the dark period,, there was a significant difference between genotype (unpaired *t*-test, *t*(14) = 2.195, *P* = 0.045). For the duration of REM episodes during the dark period,, there was no significant difference between genotype (unpaired *t*-test, *t*(14) = 1.498, *P* = 0.156).

**Figure 3i.** *n* = 8 cKO treated with saline (5 females and 3 males), and 9 cKO treated with saline (5 females and 4 males) at the age of 12 months. Data from females and males were pooled, as neither main effects of sex nor interaction effects between sex and genotype were detected (three-way ANOVA, main effects of sex, *F*(1, 28) = 0.0395, *P* = 0.844, sex × genotype interaction, *F*(1, 28) = 2.12, *P* = 0.157). There was significant genotype × treatment interaction (two-way ANOVA, genotype × treatment interaction, *F*(1, 32) = 8.58, *P* = 0.00623) Therefore, simple main effects of genotype was tested at each group, and a simple main effect of genotype was detected at MK-801 treated group (one-way ANOVA, *F*(1, 32) = 17.1, *P* = 0.000241). Simple main effects of treatment was detected at control and cKO (one-way ANOVA, control, *F*(1, 32) = 53.3, *P* < 0.001; cKO, *F*(1, 32) = 8.26, *P* = 0.00715).

**Figure 4a.** [*n* = 6 control and 6 mutant males] × 2 days (4–6 months-old). There was a significant effect of genotype at the following frequency range (unpaired *t*-test). Wake; 0.5–3 Hz, *P* = 0.00385; 4–8 Hz, *P* = 0.00644; 8–12 Hz, *P* = 0.00459; 13–30 Hz, *P* = 0.000521; 30–45 Hz, *P* = 0.00286. NREM; 30–45 Hz, *P* = 0.0138; 55–80 Hz, *P* = 0.0273. REM; 0.5–3 Hz, *P* = 0.0308; 13–30 Hz, *P* = 0.000520; 30–45 Hz, *P* = 0.00216.

**Figure 4b. [***n* = 6 control and 6 mutant males] × 2 days (4–6 months-old). There was a significant effect of genotype at the following frequency range (unpaired *t*-test). Wake; 0.5–3 Hz, *P* = 0.0100; 4–8 Hz, *P* = 0.0211; 8–12 Hz, *P* = 0.0141; 13–30 Hz, *P* = 0.00242; 30–45 Hz, *P* = 0.0144. NREM; 30–45 Hz, *P* = 0.0302. REM; 0.5–3 Hz, *P* = 0.0335; 13–30 Hz, *P* = 0.000654; 30–45 Hz, *P* = 0.0242.

**Figure 5c. Left.** *n* = 12 and 12 males for each group (3 months-old). There was significant difference between genotype (Welch’s *t*-test, *t*(16.4) = 3.09, *P* = 0.00683).

**Figure 5c. Right.** *n* = 12 and 12 males for each group (3 months-old). There was no significant difference between genotype (unpaired *t*-test, *t*(22) = 1.45, *P* = 0.162).

**Figure 5d.** *n* = 6 control-tRFP, 8 cKO-tRFP, and 9 cKO-rescue males. There was no significant interaction between genotype and time, and there was a significant main effect of genotype (mixed between-within subjects ANOVA, genotype × time interaction, *F*(6, 60) = 1.96, *P* = 0.0850, main effects of genotype, *F*(2, 20) = 4.43, *P* = 0.0256). Tukey's *post-hoc* test showed significant difference between genotype at indicated points in the graph.

**Figure 5e.** *n* = 15 control-tRFP (8 females and 7 males), 13 cKO-tRFP (5 females and 8 males), and 14 cKO-rescue (8 females and 6 males), 4 months-old. Data from females and males were pooled, as neither main effects of sex nor interaction effects between sex and genotype were detected (mixed between-within subjects ANOVA, main effects of sex, *F*(1, 36) = 0.162, *P* = 0.390, sex × genotype interaction, *F*(2, 23) = 0.227, *P* = 0.798). There was a significant main effect of genotype (one-way ANOVA, *F*(2, 39) = 11.4, *P* = 0.000127). Tukey's *post-hoc* test showed significant difference between genotype at indicated points in the graph.

**SI Refferences**

1. Tsien JZ, Huerta PT, Tonegawa S. The essential role of hippocampal CA1 NMDA receptor-dependent synaptic plasticity in spatial memory. *Cell* 1996; **87**(7)**:** 1327-1338.

2. Madisen L, Zwingman TA, Sunkin SM, Oh SW, Zariwala HA, Gu H *et al.* A robust and high-throughput Cre reporting and characterization system for the whole mouse brain. *Nature neuroscience* 2010; **13**(1)**:** 133-140.

3. Kobayashi Y, Sano Y, Vannoni E, Goto H, Suzuki H, Oba A *et al.* Genetic dissection of medial habenula-interpeduncular nucleus pathway function in mice. *Front Behav Neurosci* 2013; **7:** 17.

4. Belforte JE, Zsiros V, Sklar ER, Jiang Z, Yu G, Li Y *et al.* Postnatal NMDA receptor ablation in corticolimbic interneurons confers schizophrenia-like phenotypes. *Nat Neurosci* 2010; **13**(1)**:** 76-83.

5. Cancino GI, Yiu AP, Fatt MP, Dugani CB, Flores ER, Frankland PW *et al.* p63 Regulates adult neural precursor and newly born neuron survival to control hippocampal-dependent Behavior. *J Neurosci* 2013; **33**(31)**:** 12569-12585.

6. Hayashi Y, Kashiwagi M, Yasuda K, Ando R, Kanuka M, Sakai K *et al.* Cells of a common developmental origin regulate REM/non-REM sleep and wakefulness in mice. *Science (New York, NY)* 2015; **350**(6263)**:** 957-961.

7. Pitychoutis PM, Belmer A, Moutkine I, Adrien J, Maroteaux L. Mice Lacking the Serotonin Htr2B Receptor Gene Present an Antipsychotic-Sensitive Schizophrenic-Like Phenotype. *Neuropsychopharmacology* 2015; **40**(12)**:** 2764-2773.

8. Zhang F, Gradinaru V, Adamantidis AR, Durand R, Airan RD, de Lecea L *et al.* Optogenetic interrogation of neural circuits: technology for probing mammalian brain structures. *Nature protocols* 2010; **5**(3)**:** 439-456.

9. Parker JG, Beutler LR, Palmiter RD. The contribution of NMDA receptor signaling in the corticobasal ganglia reward network to appetitive Pavlovian learning. *J Neurosci* 2011; **31**(31)**:** 11362-11369.

10. Rohr UP, Wulf MA, Stahn S, Steidl U, Haas R, Kronenwett R. Fast and reliable titration of recombinant adeno-associated virus type-2 using quantitative real-time PCR. *J Virol Methods* 2002; **106**(1)**:** 81-88.

**SI Figures and Legends**


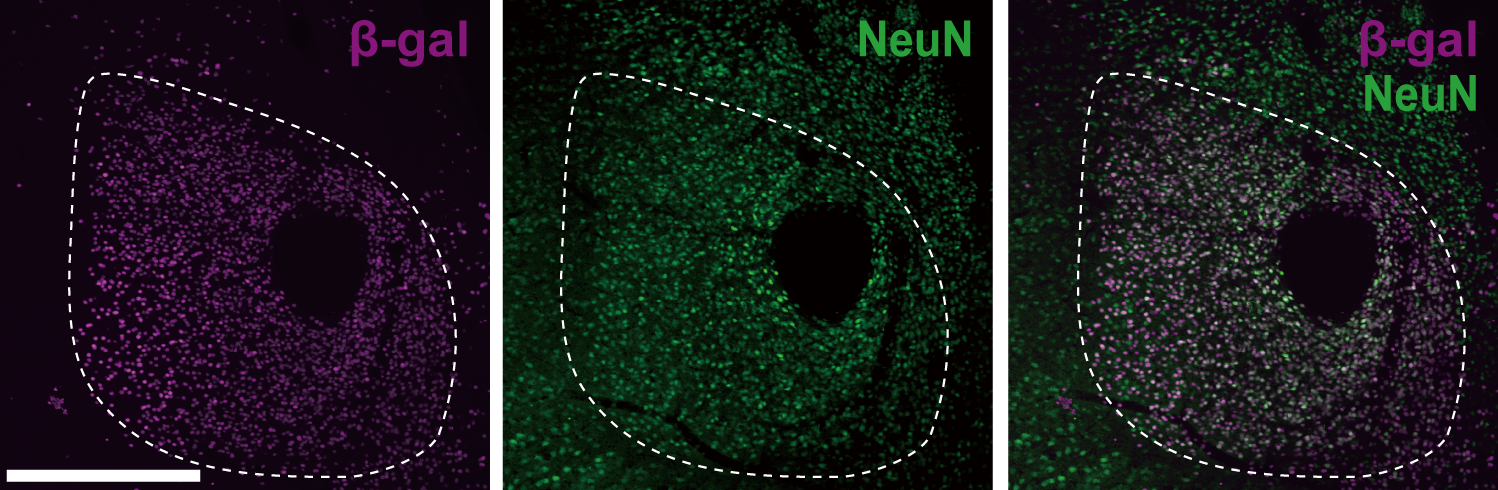


**Supplementary Figure 1.** Representative images of immunohistochemistry in the ILN-Cre; Rosa-NLSLacZ double transgenic mouse. Cre recombination, resulting in β-galactosidase (β-gal) expression (magenta), occurred in 87.3 ± 2.24% (*n* = 2 mice at 1.5 months of age,) of NeuN positive neurons (green) in the ILN. Scale bars: 500 μm.


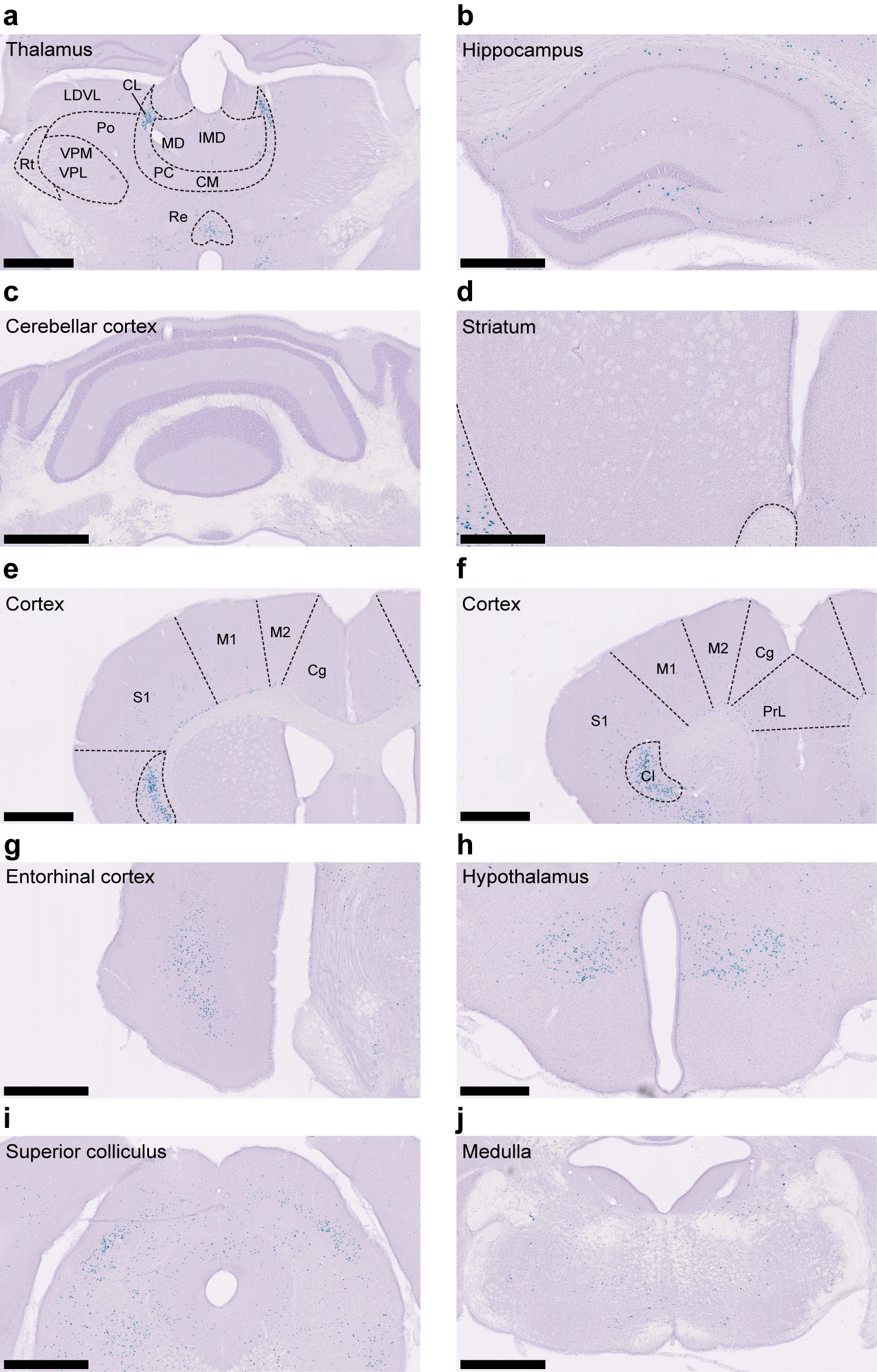
**Supplementary Figure 2.** Representative images (from 4 mice) of the spatial distribution of Cre recombinase activity in coronal sections from an ILN-Cre::Rosa-NLSLacZ double-transgenic mouse stained with X-gal (blue) and hematoxylin (purple). (a) LacZ-positive cells are restricted to a subregion of the anterior ILN, the centrolateral (CL) thalamic nucleus, with a few LacZ-positive cells in the mediodorsal (MD), central medial (CM), reticular (Rt), ventral posteromedial (VPM), ventral posterolateral (VPL), and posterior (Po) thalamic nuclei. The reuniens (Re) thalamic nucleus contained a few LacZ-positive cells. (b) A few LacZ-positive cells were detected in the hippocampus. (c, d) No LacZ-positive cells were detected in the cerebellar cortex and the striatum. (e, f, g) A few LacZ-positive cells were detected in the cingulate (Cg), prelimbic (PrL), motor (M), somatosensory (S), and entorhinal cortices. The claustrum (Cl) contained a substantial number of LacZ-positive cells. (h) A few LacZ-positive cells were also detected in the hypothalamus. (i, j) Some LacZ-positive cells were scattered in the superior colliculus and medulla. (a, c, e, f, g, i, j) Scale bars, 1 mm. (b, d, h) Scale bars, 0.5 mm.


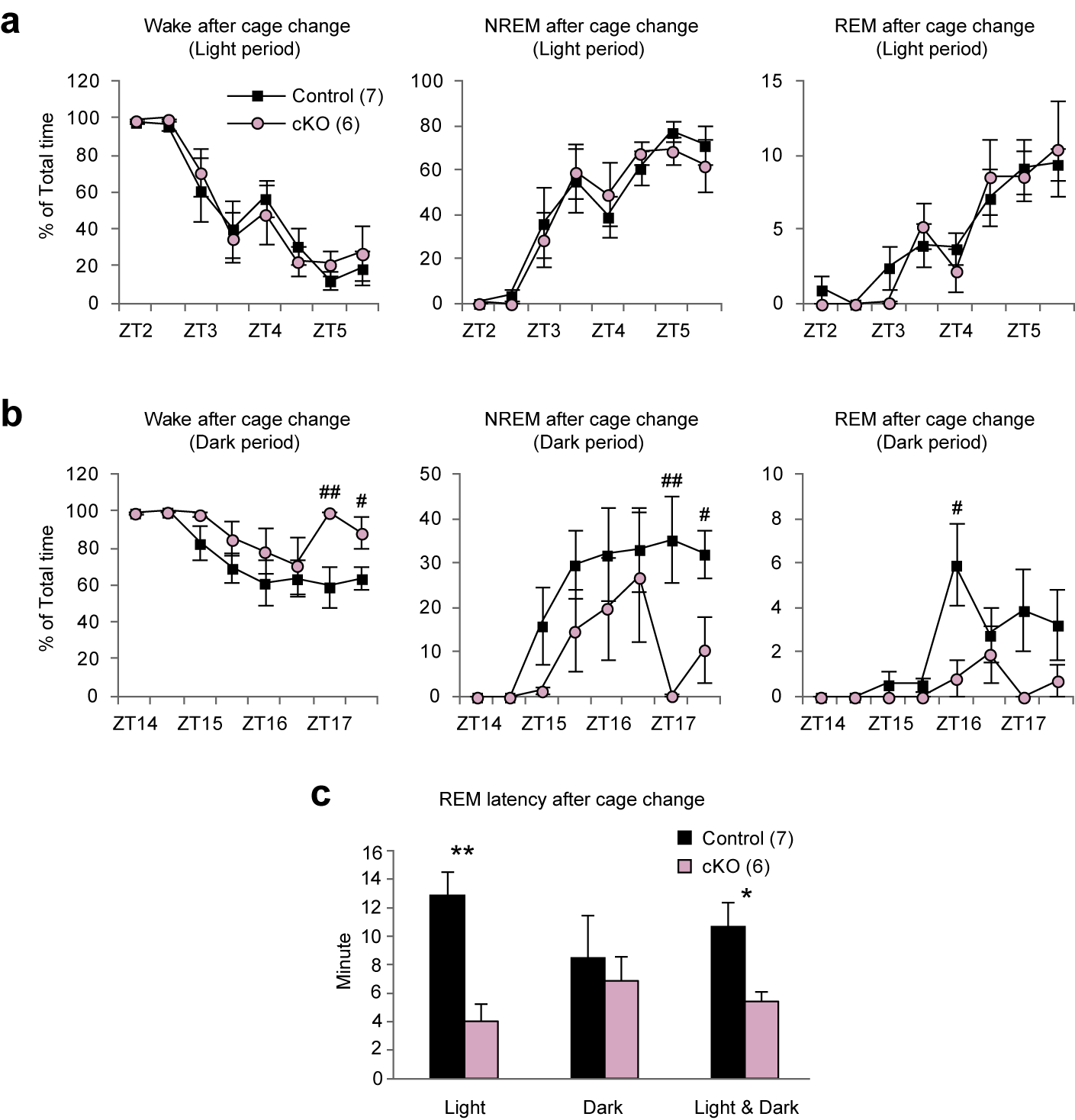


**Supplementary Figure 3.** The cKO mice exhibited enhanced arousal during the dark period. For cage change experiments, EEG/EMG recorded mice underwent a cage change at Zeitgeber time (ZT) 2, during the light phase, and after 36 hrs, underwent the second cage change at ZT14, during the dark phase. (a, b) Sleep/wake patterns following cage change either during the light period (a) or the dark period (b) in control mice (*n* = 7) and cKO mice (*n* = 6). (c) The REM latency, defined as the time elapsing from sleep onset to the beginning of first episode of REM sleep, was decreased in the cKO mice during the light phase. #*P* < 0.05, ##*P* < 0.01 [one-way ANOVA]. **P* < 0.05, ***P* < 0.01 [unpaired *t*-test]. All error bars represent SEM. (a) *n* = 7 control and 6 mutant males. For wake, NREM, and REM after cage change (light period), simple main effect of genotype at each time point was tested, and no significant effect was detected at any time points (one-way ANOVA). (b) *n* = 7 control and 6 mutant males. For wake after cage change (dark period), simple main effect of genotype at each time point was tested, and a significant effect was detected at the following time points (one-way ANOVA: (at ZT17) *F*(1, 11) = 11.629, *P* = 0.006, (at ZT17.5) *F*(1, 11) = 5.823, *P* = 0.034). For NREM after cage change (dark period), simple main effect of genotype at each time point was tested, and a significant effect was detected at the following time points (one-way ANOVA: (at ZT17) *F*(1, 11) = 10.948, *P* = 0.007, (at ZT17.5) *F*(1, 11) = 5.548, *P* = 0.038). For REM after cage change (dark period), simple main effect of genotype at each time point was tested, and a significant effect was detected at the following time points (one-way ANOVA: (at ZT16) *F*(1, 11) = 5.612, *P* = 0.037). (c) *n* = 7 control and 6 mutant males. For REM latency during the light phase, there was a significant difference in the REM latency during the light phase (unpaired *t*-test, *t*(11) = 4.275, *P* = 0.001). For REM latency during the dark phase, there was no significant difference between genotype (unpaired *t*-test, *t*(11) = 0.442, *P* = 0.653). For REM latency during the whole day, there was a significant difference difference between genotype (unpaired *t*-test, *t*(7.667) = 2.923, *P* = 0.020).


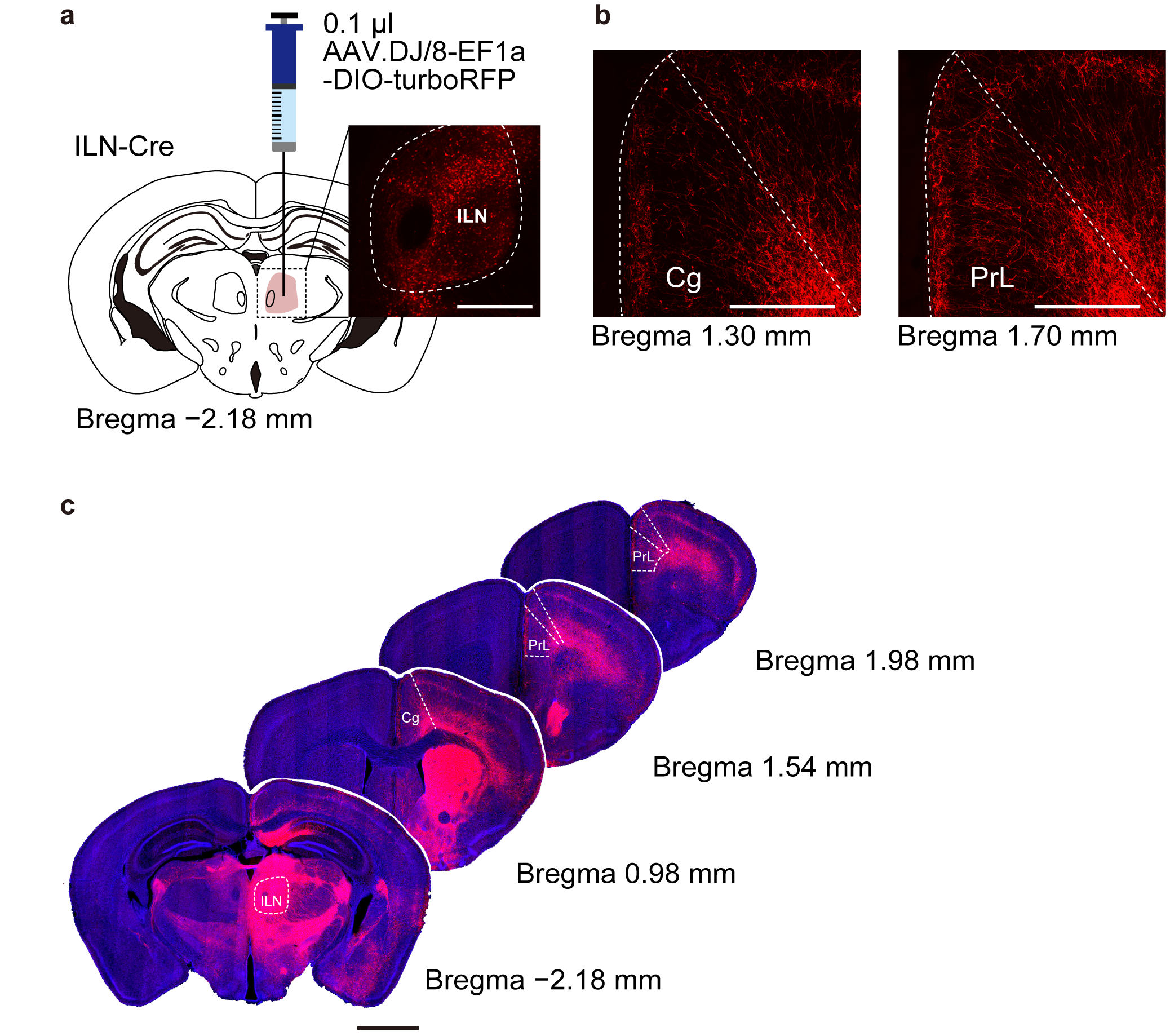


**Supplementary Figure 4.** Anatomical characterization of the ILN cells using viral vectors. (a) ILN-Cre mice were injected unilaterally with 0.1 μl AAV.DJ/8- EF1a-DIO-turboRFP. TurboRFP expression was detected in the ILN. Scale bar, 0.5 mm. (b) TurboRFP-positive axon varicosities were detected in the prefrontal and anterior cingulate cortices. Scale bar, 0.5 mm. (c) Representative images of diffuse projections form the ILN into multiple cortical areas and striatum. Scale bar, 2 mm.
